# Polarized Axonal Surface Expression of Neuronal KCNQ Potassium Channels Is Regulated by Calmodulin Interaction with KCNQ2 Subunit

**DOI:** 10.1371/journal.pone.0103655

**Published:** 2014-07-31

**Authors:** John P. Cavaretta, Kaitlyn R. Sherer, Kwan Young Lee, Edward H. Kim, Rodal S. Issema, Hee Jung Chung

**Affiliations:** 1 Department of Molecular and Integrative Physiology, University of Illinois at Urbana-Champaign, Urbana, Illinois, United States of America; 2 Neuroscience Program, University of Illinois at Urbana-Champaign, Urbana, Illinois, United States of America; University of Texas Health Science Center, United States of America

## Abstract

KCNQ potassium channels composed of KCNQ2 and KCNQ3 subunits give rise to the M-current, a slow-activating and non-inactivating voltage-dependent potassium current that limits repetitive firing of action potentials. KCNQ channels are enriched at the surface of axons and axonal initial segments, the sites for action potential generation and modulation. Their enrichment at the axonal surface is impaired by mutations in KCNQ2 carboxy-terminal tail that cause benign familial neonatal convulsion and myokymia, suggesting that their correct surface distribution and density at the axon is crucial for control of neuronal excitability. However, the molecular mechanisms responsible for regulating enrichment of KCNQ channels at the neuronal axon remain elusive. Here, we show that enrichment of KCNQ channels at the axonal surface of dissociated rat hippocampal cultured neurons is regulated by ubiquitous calcium sensor calmodulin. Using immunocytochemistry and the cluster of differentiation 4 (CD4) membrane protein as a trafficking reporter, we demonstrate that fusion of KCNQ2 carboxy-terminal tail is sufficient to target CD4 protein to the axonal surface whereas inhibition of calmodulin binding to KCNQ2 abolishes axonal surface expression of CD4 fusion proteins by retaining them in the endoplasmic reticulum. Disruption of calmodulin binding to KCNQ2 also impairs enrichment of heteromeric KCNQ2/KCNQ3 channels at the axonal surface by blocking their trafficking from the endoplasmic reticulum to the axon. Consistently, hippocampal neuronal excitability is dampened by transient expression of wild-type KCNQ2 but not mutant KCNQ2 deficient in calmodulin binding. Furthermore, coexpression of mutant calmodulin, which can interact with KCNQ2/KCNQ3 channels but not calcium, reduces but does not abolish their enrichment at the axonal surface, suggesting that apo calmodulin but not calcium-bound calmodulin is necessary for their preferential targeting to the axonal surface. These findings collectively reveal calmodulin as a critical player that modulates trafficking and enrichment of KCNQ channels at the neuronal axon.

## Introduction

Neuronal KCNQ channels are voltage-dependent potassium (K^+^) channels composed mostly of K_v_7.2/KCNQ2 and K_v_7.3/KCNQ3 subunits [1], which are found throughout the nervous system including the hippocampus [2]–[6]. They are activated at the sub-threshold potentials of action potential generation. Thus, they allow the firing of a single action potential, but effectively inhibit repetitive firing of action potentials [1]. Originally named “M-channels”, their inhibition by muscarinic agonists profoundly increases action potential firing [7]. In addition, KCNQ channels regulate resting membrane potential and contribute to spike frequency adaptation, spike afterdepolarization, and spike afterhyperpolarization [8]–[13]. Consistent with their ability to suppress burst and spontaneous firing of action potentials [10], [14], mutations in KCNQ2 and KCNQ3 cause benign familial neonatal convulsions (BFNC) that are variably associated with drug-resistant epilepsy, Rolandic epilepsy, mental retardation, developmental delay, and peripheral nerve hyperexcitability [15]. Notably, inhibition of KCNQ current in the neonatal brain increases spontaneous seizures and seizure susceptibility in mice [8], whereas the KCNQ channel opener ezogabine/retigabine is used as an anti-epileptic drug [16].

Axonal rather than somatic KCNQ channels have been shown to suppress action potential firing in hippocampal CA1 neurons by regulating action potential threshold and resting membrane potential [14], [17]. Computer modeling based on electrophysiological data has predicted that their axonal conductance must be 3–5 times greater than their somatic conductance in order to prevent spontaneous action potential firing in a “near-realistic” model of the CA1 pyramidal cell [14]. Indeed, KCNQ channels are enriched at the axonal surface with the highest concentration in the axonal initial segment (AIS) [18], the critical site for action potential initiation and modulation [19]. Localization of KCNQ channels at the AIS requires KCNQ2 and KCNQ3 interaction with ankyrin-G [6], [18], [20], an essential component of the AIS that maintains neuronal axon versus dendrite polarity [21]. Disrupting the normal channel localization at the AIS causes bursting and epileptiform activity [14]. Importantly, BFNC mutations in the cytoplasmic carboxy (C)-terminal tail of KCNQ2 decrease surface density of KCNQ channels at the AIS and distal axons [18]. Despite the significant implications of axonal KCNQ conductance in neuronal excitability, it is unclear how enrichment of KCNQ channels at the axonal surface is achieved.

**Figure 1:**
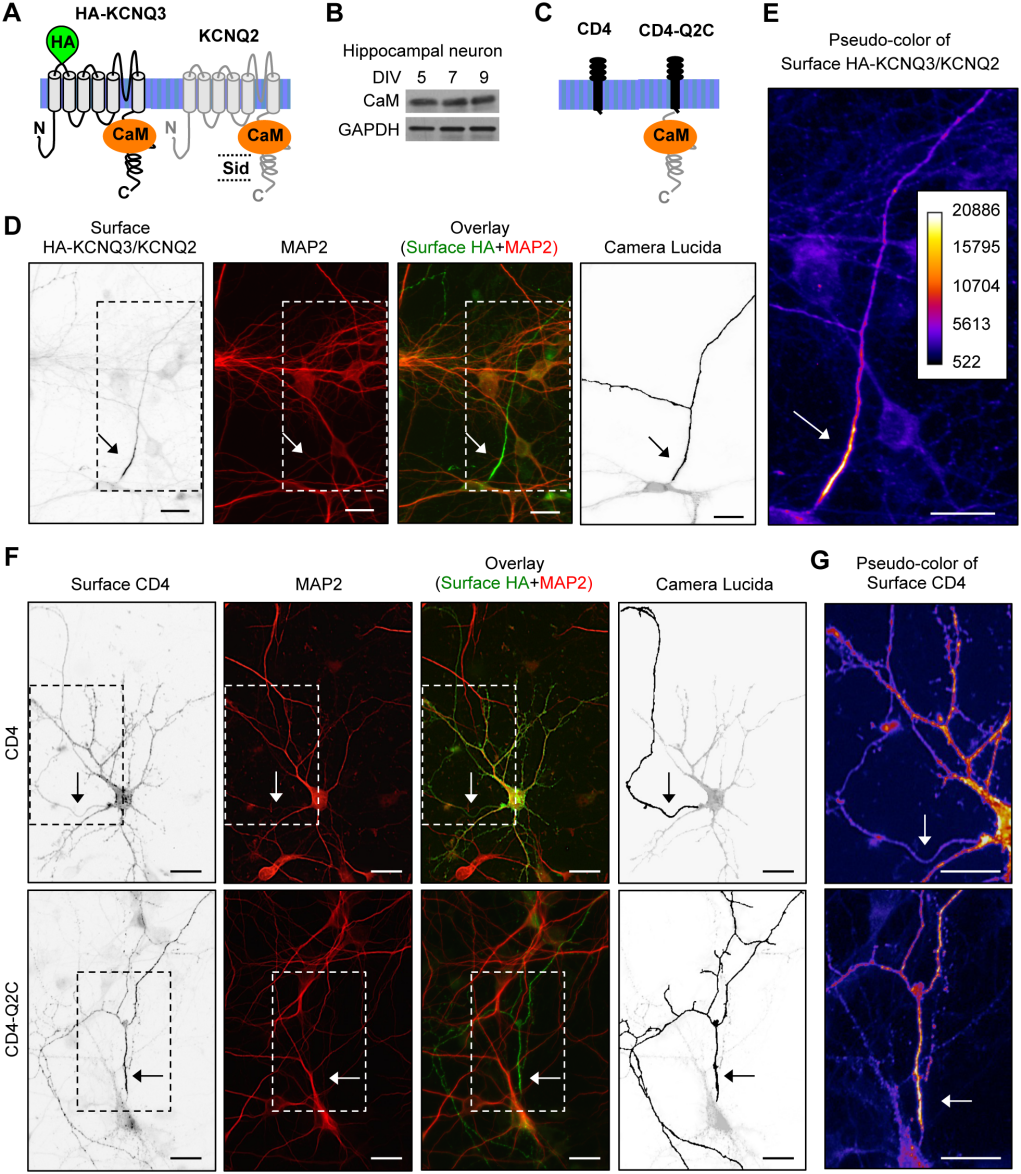
Enrichment of HA-KCNQ3/KCNQ2 channels and CD4-Q2C at the axonal surface. Schematic drawings (not to scale) of a human KCNQ2 subunit (accession #Y15065), including the subunit interaction domain (Sid, amino acids 580–623) [52], [53], and CaM-binding domain (amino acids 323–579) [22], [23]. (B) Immunoblot analysis of CaM in cultured rat hippocampal neurons at 5–9 days in vitro (DIV). Glyceraldehyde-3-phosphate dehydrogenase (GAPDH) served as a loading control. (C) Schematic drawings (not to scale) of a human CD4 protein, and CD4 fused to KCNQ2 C-terminal tail (CD4-Q2C, [18]). (D) Surface immunostaining of hippocampal neurons (DIV 7) transfected with KCNQ2 and KCNQ3 containing an extracellular hemagglutinin (HA) epitope (HA-KCNQ3). Neuronal soma and dendrites were visualized by immunostaining for MAP2. Surface HA-KCNQ3/KCNQ2 channels are enriched on a MAP2-negative neurite that originates directly from the soma. (E) Pseudo-color image of the inset in Fig. 1D displays differences in the surface HA intensity. Surface HA-KCNQ3/KCNQ2 channels are enriched at the initial segment of an axon. (F) Surface immunostaining of hippocampal neurons (DIV 8) transfected with CD4, or CD4-Q2C. (G) Pseudo-color images of the insets in Fig. 1F display differences in the surface CD4 intensity. Fusion of KCNQ2 C-terminal tail enriches CD4 at the axonal surface. Camera lucida drawings of the neuronal images (D, F) show the soma and dendrites (gray) and an axon (black). Arrows mark the main axon. Scale bars are 20 µm.

The axon targeting signals have been shown to reside in the first 256 amino acid residues of the KCNQ2 C-terminal tail [18]. This region contains two helical domains (helices A and B) that bind to calcium (Ca^2+^) sensor, calmodulin (CaM) [22], [23] (Fig. 1A, 2A). Helix A contains the consensus CaM binding IQ motif whereas helix B mediates Ca^2+^-dependent CaM binding [22]–[25]. Since CaM interaction with KCNQ2 is required for functional expression of KCNQ channels in non-neuronal cells [22], [24], [26], we hypothesized that CaM regulates preferential targeting of KCNQ channels to the axonal surface. By utilizing a dominant-negative mutant CaM that is unable to bind Ca^2+^ [27] or mutations that block or reduce CaM binding [24], [26], we show that CaM interaction with the IQ motif of KCNQ2 is required for exit of KCNQ channels from the endoplasmic reticulum (ER), and their subsequent enrichment at the axonal plasma membranes in cultured hippocampal neurons.

**Figure 2:**
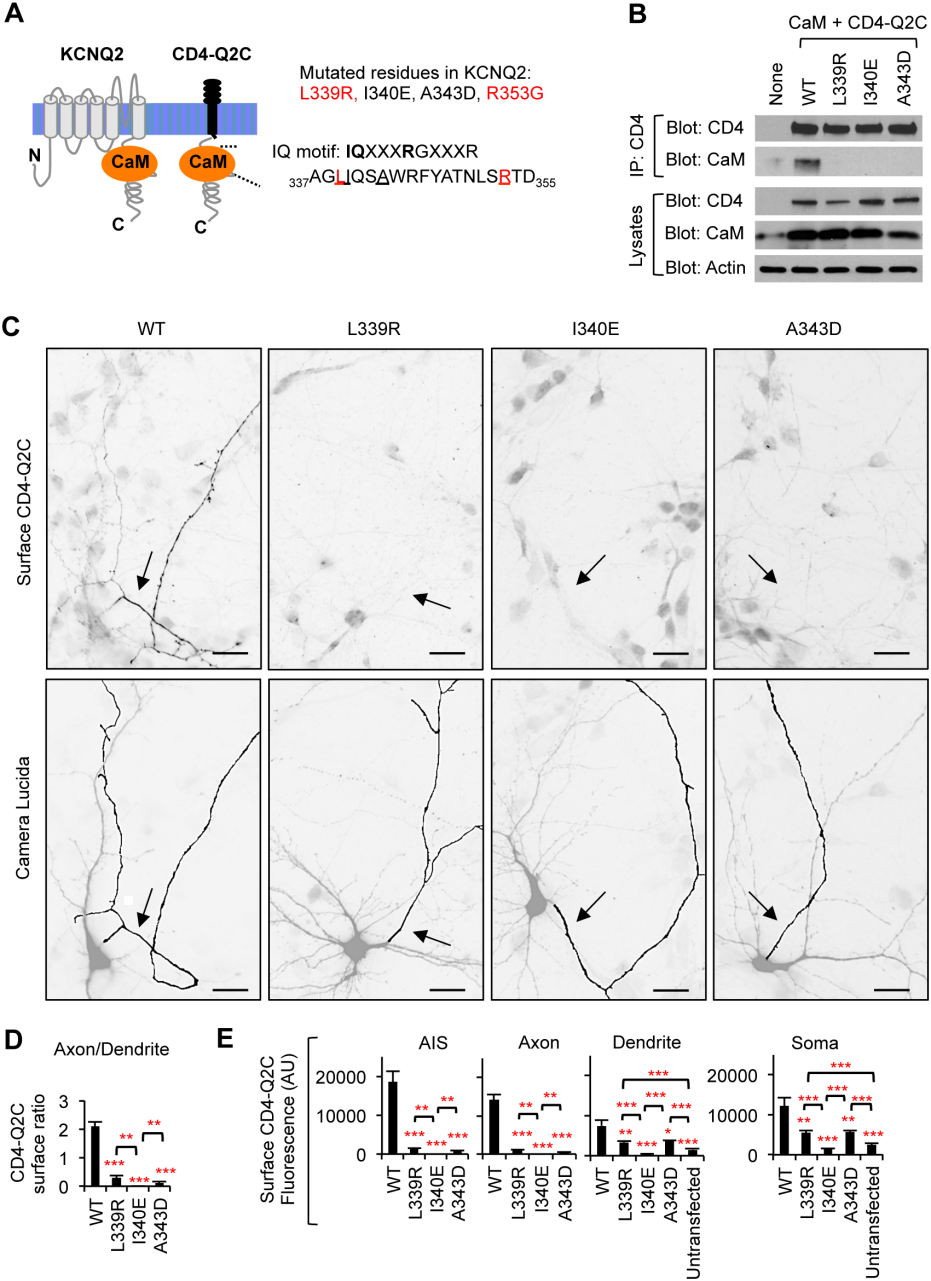
Mutations in IQ motif abolish axonal enrichment of surface CD4-Q2C. (A) Schematic drawings (not to scale) of a human KCNQ2 subunit (accession #Y15065) and CD4-Q2C showing CaM-binding domain. The amino acids in bold text are critical residues in the CaM-binding consensus IQ motif in helix A. Mutations in the underlined amino acids have been shown to abolish (L339R, I340E, and A343D) or moderately decrease (R353G) CaM interaction with KCNQ2 [24], [26]. Mutations in the amino acids colored red are associated with BFNC [43]. (B) Lysates from HEK293T cells expressing CaM and CD4-Q2C wild-type (WT) or mutant proteins (L339R, I340E, and A343D) were subjected to immunoprecipitation (IP) with the CD4 antibody. Immunoprecipitation and total cell lysates were analyzed by immunoblotting for CD4 and CaM. β-actin served as a loading control. The L339R, I340E, and A343D mutations abolished co-immunoprecipitation of CaM with CD4-Q2C. (C) Surface immunostaining of CD4-Q2C WT or mutant proteins in hippocampal neurons (DIV 7–8). Camera lucida drawings (lower) of the inverted images of surface CD4-Q2C (upper) show the soma and dendrites (gray) and an axon (black). The axon was identified by immunostaining for the AIS marker phospho IκBα Ser32 (14D4) in the neurons cotransfected with GFP, which allows visualization of all neurites (Figure S3). Arrows indicate the AIS. Scale bars are 20 µm. The L339R, I340E, and A343D mutations abolished surface expression of CD4-Q2C at the AIS and distal axon. (D) The surface “Axon/Dendrite” ratios of CD4-Q2C were reduced to nearly 0 by L339R, I340E, and A343D mutations. (E) Background subtracted, mean intensity of surface CD4 fluorescence in the AIS, distal axons, soma, and major dendrites. AU, arbitrary unit. The sample number for each construct used in (D, E) was as follows: WT (n = 18), L339R (n = 20), I340E (n = 12), A343D (n = 25), and untransfected (n = 20). Ave ± SEM (*p<0.05, **p<0.01, ***p<0.001).

## Materials and Methods

### Materials

Antibodies used in immunocytochemistry include mouse anti-ankyrin G (Neuromab, 75–146), rabbit anti-phospho IκBα Ser32 (14D4) (Cell Signaling, 2859), rabbit anti-MAP2 antibody (Millipore, AB5622), chicken anti-MAP2 antibody (Abcam, ab5392), Alexa 488-conjugated and unconjugated mouse anti-CD4 antibody (Invitrogen/Molecular Probes, MHCD0420 and MHCD0400), Alexa 488-conjugated and unconjugated mouse anti-HA monoclonal antibody (Covance Research Products, A488-101L and MMS-101P), rabbit anti-HA monoclonal antibody (Cell Signaling, 3724), rabbit anti-CaM antibody (Cell signaling, 4830), donkey anti-mouse secondary antibody (The Jackson Laboratory, 715-005-151), and Alexa488-, Alexa594-, Alexa647-, Alexa680-, and Alexa700-conjugated secondary antibodies (Invitrogen/Molecular Probes, A21202, A21206, A21203, A21207, A21449, A10038, A10043, A21036, and A21038). Reagents used in immunoprecipitation and western blotting include human embryonic kidney (HEK) 293T cell lines (ATCC, CRL-11268) and antibodies including anti-CD4 (Santa Cruz Biotech, sc-13573), anti-HA (Covance, MMS-101P, Cell Signaling, 3724), anti-KCNQ2 (Alamone, APC-050; Neuromab, N26A/23), anti-CaM (Novus Biologicals, NB110-55649; Santa Cruz, SC137079; Cell Signaling, 4830), anti-HA, anti-β-actin, and anti-glyceraldehyde-3-phosphate dehydrogenase (GAPDH) antibodies (Cell Signaling, 3724, 4967, and 2118). Reagents used in pulse-chase experiment include brefeldin A (Sigma, B7651).

### DNA constructs and Mutagenesis

The plasmids pcDNA3 containing CD4 or CD4 fused to KCNQ2 C-tail (CD4-Q2C), CD4-GFP-KDEL, KCNQ2 (accession #, Y15065), and KCNQ3 tagged with an extracellular hemagglutinin (HA) epitope (HA-KCNQ3) have been described [18], [28], [29]. Plasmids pEGFPN1 (Invitrogen) and pcDNA3-containing mCherry were gifts from Dr. Lily Jan (University of California San Francisco). Plasmids pJPA7 containing wild-type CaM and Ca^2+^-insensitive CaM mutant (CaM1234) were gifts from Dr. John Adelman (Oregon Health and Science University, Portland, OR) [27]. Mutations in the IQ consensus residues of KCNQ2 (I340E, A343D), and BFNC mutations (L339R, R353G) were generated using the QuikChange II XL Site-Directed Mutagenesis Kit (Agilent), and they were verified by sequencing the entire cDNA construct. The following oligonucleotides were used for mutagenesis: L339R (sense-^5′^CGG CAG CAG GCA GGA TCC AGT CGG C^3′^, antisense-^5′^ GCC GAC TGG ATC CTG CCT GCT GCC G^3′^), I340E (sense-^5′^ CAG CAG GCC TGG AAC AGT CGG CCT G^3′^, antisense-^5′^ CAG GCC GAC TGT TCC AGG CCT GCT G^3′^), A343D (sense-^5′^ CTG ATC CAG TCG GAC TGG AGA TTC TAC GC^3′^, antisense-^5′^GCG TAG AAT CTC CAG TCC GAC TGG ATC AG^3′^), R353G (sense-^5′^CAC CAA CCT CTC GGG CAC AGA CCT GCA C^3′^, antisense-^5′^GTG CAG GTC TGT GCC CGA GAG GTT GGT G^3′^). The underlined bases indicate amino acid substitution mutations in the sense and antisense sequences.

### Experimental animals

All procedures involving animals were reviewed and approved by the Institutional Animal Care and Use Committee at the University of Illinois Urbana-Champaign in accordance with the guidelines of the U.S. National Institutes of Health (Protocols 10199, 12240). The timed-pregnant Sprague-Dawley rats were purchased from Charles River. To minimize stress and discomfort, the timed-pregnant rats were euthanized by inhalation of carbon dioxide followed by decapitation. The 18–19 day embryonic rats were quickly removed by caesarian section and decapitated. The hippocampi of embryos were dissected in ice-cold slice dissection solution containing (in mM): 10 HEPES, 82 Na_2_SO_4_, 30 K_2_SO_4_, 10 Glucose, 5 MgCl_2_ (pH 7.4).

### Neuronal Cell Culture and Transfection

Primary dissociated hippocampal cultures were prepared from hippocampi of 18–19 day embryonic rats as described [30] with the following modifications: Neurons were plated on 12 mm glass coverslips (Warner Instruments, 10^5^ cells per coverslip), or 30 mm cell culture dishes (BD Biosciences, 7×10^5^ cells per dish) coated with poly L-lysine (0.1 mg/mL). Neurons were maintained in neurobasal medium supplemented with B27 extract, 200 mM L-glutamine, and 100 U/mL penicillin and streptomycin in a cell culture incubator (37°C, 5% CO_2_) for 5–9 days *in vitro*. At 5–6 days *in vitro*, neurons were transfected with plasmids (total 0.8 µg) using Lipofectamine LTX (Invitrogen) according to manufacturer’s protocol.

### Immunocytochemistry

Surface and permeabilized immunostaining were performed at room temperature at 48 hr post transfection (7–8 days *in vitro*) as described [18] with the following modifications. For surface immunostaining of CD4 and CD4-Q2C proteins, neurons were washed once with artificial cerebral spinal fluid (ACSF) solution containing (in mM): 10 HEPES, 150 NaCl, 3 KCl, 2 CaCl_2_, 10 Dextrose (pH 7.4). Neurons were fixed in 4% paraformaldehyde/4% sucrose in Phosphate buffered saline (PBS) for 7 min, washed with PBS, blocked with 10% normal donkey serum in PBS for 1 hr, and incubated with Alexa 488-conjugated mouse anti-CD4 antibody (1∶500 dilution) in 3% normal donkey serum in PBS at 4°C overnight. After the PBS wash, the neurons were incubated with donkey anti-mouse secondary antibody (0.13 mg/mL) for 2 hr and fixed again for 15 min. The neurons were permeabilized with 0.2% Triton X-100 in PBS for 10 min, and incubated with rabbit anti-MAP2 antibody (1∶2000 dilution) alone, or with mouse anti-CD4 antibody (1∶500 dilution) to label intracellular CD4 proteins and rabbit anti-MAP2 antibody (1∶2000 dilution), or rabbit anti-phospho IκBα Ser32 (14D4) antibody (1∶400) and chicken anti-MAP2 antibody (1∶5000 dilution). After the PBS wash, the neurons were incubated with Alexa594- and Alexa647- or Alexa700-conjugated secondary antibodies (1∶200 dilution) in 3% normal donkey serum in PBS. After the PBS wash, the coverslips were mounted using Fluorogel anti-fade mounting medium (Electron Microscopy Sciences). For surface immunostaining of CD4-Q2C wild-type or mutant proteins in the neurons cotransfected with GFP, the neurons were incubated with mouse anti-CD4 antibody (1∶500 dilution) without permeabilization at 4°C overnight, followed by incubation with Alexa 594-conjugated secondary antibodies (1∶200 dilution) without permeabilization for 2 hr. The neurons were then fixed, permeabilized, and incubated with rabbit anti-phospho IκBα Ser32 (14D4) antibody (1∶400) or rabbit anti-MAP2 antibody (1∶2000 dilution), followed by incubation with Alexa 700-conjugated secondary antibodies (1∶200 dilution). For permeabilized immunostaining of CD4-Q2C proteins, the neurons were fixed, permeabilized, and incubated overnight at 4°C with mouse anti-CD4 antibody and rabbit anti-phospho IκBα Ser32 (14D4) antibody or chicken (or rabbit) anti-MAP2 antibody.

For surface immunostaining of HA-KCNQ3/KCNQ2 proteins, the neurons were incubated overnight at 4°C with Alexa 488-conjugated mouse anti-HA antibody (1∶500 dilution) without permeabilization. After permeabilization, the neurons were incubated with rabbit anti-MAP2 antibody (1∶2000 dilution) alone or with rabbit anti-HA antibody (to label total HA-KCNQ3 proteins, 1∶500 dilution) and chicken anti-MAP2 antibody. For surface immunostaining of HA-KCNQ3/KCNQ2 proteins in the neurons transfected with GFP, the neurons were incubated with mouse anti-HA antibody (1∶500 dilution) followed by incubation with Alexa 594-conjugated secondary antibodies (1∶200 dilution) without permeabilization. The neurons were then fixed, permeabilized, and incubated with rabbit anti-phospho IκBα Ser32 (14D4) antibody (1∶400) or rabbit anti-MAP2 antibody (1∶2000 dilution), followed by incubation with Alexa700-conjugated secondary antibodies (1∶200 dilution). For surface immunostaining of HA-KCNQ3/KCNQ2 proteins in the neurons transfected with pcDNA3, CaM WT, or CaM1234, neurons were first immunostainined for surface HA as described above. The neurons were then fixed, permeabilized, and incubated with rabbit anti-CaM antibody (1∶200 dilution) and mouse anti-MAP2 antibody (1∶2000 dilution), followed by incubation with Alexa488- and Alexa700-conjugated secondary antibodies (1∶200 dilution). For permeabilized immunostaining of HA-KCNQ3/KCNQ2 proteins, the neurons were fixed, permeabilized, and incubated overnight at 4°C with mouse anti-HA antibody, rabbit anti-phospho IκBα Ser32 (14D4) antibody, and chicken anti-MAP2 antibody. For permeabilized immunostaining of HA-KCNQ3/KCNQ2 and CD4-GFP-KDEL, the neurons were incubated with mouse anti-HA antibody, and rabbit anti-MAP2 antibody (1∶1000 dilution). For permeabilized immunostaining of CaM, the neurons were incubated with rabbit anti-CaM antibody, mouse anti-ankyrin-G antibody (1∶200 dilution), and chicken anti-MAP2 antibody.

### Pulse-Chase Assay after removal of brefeldin-A

Pulse-Chase assay after brefeldin-A (BFA) removal was performed as described [31] with the following modifications: 30 min post transfection with CD4-Q2C or HA-KCNQ3/KCNQ2, half of the original medium per well was saved, and the transfected neurons were incubated with vehicle control (0.1% ethanol), or BFA (0.75 µg/ml) in the remaining medium and returned to the cell culture incubator. At 16 hr post-BFA treatment, the neurons were washed with fresh medium (37°C) and returned to the cell culture incubator with the saved original medium for 0, 1, 2, 4, 8, or 24 hr. The same experiments were repeated with the neurons co-transfected with GFP to visualize their entire neuronal morphology.

### Image Acquisition and Quantification

Fluorescence and phase contrast images of transfected neurons were viewed using a Zeiss Axiovert 200 M inverted microscope with appropriate filter sets. The transfected neurons were examined for integrity and health using fluorescence imaging of MAP2 and/or GFP staining and differential interference contrast (DIC) imaging. If the transfected neurons had beaded or broken dendrites or axons, or damaged soma, then they were excluded from analysis. High-resolution gray scale images of healthy transfected neurons were acquired using 20X, 40X, or 63X objectives with a Zeiss AxioCam HRm Camera and Axiovert software and saved as 16-bit ZVI and TIFF files. Within one experiment, the images were acquired using the same exposure time to compare the fluorescence intensity of the neurons transfected with different constructs, and stored with no further modifications.

The fluorescence intensity profiles of the soma and the major axonal and dendritic processes from the 16-bit TIFF files were quantified using ImageJ Software (National Institutes of Health, USA, http://rsb.info.nih.gov/ij) as previously described [18]. The dendrites were identified as MAP2-positive processes from the transfected neuron (Figure S1, Figure S2) or the processes that were absent for the AIS markers, phospho IκBα Ser32 (14D4) [32] or ankyrin-G [21] in the GFP-transfected neuron (Figure S3, Figure S4). The axon was identified as a MAP2-negative process from the transfected neuron (Figure S1, Figure S2) or a process that were labeled for the AIS markers in the GFP-transfected neuron (Figure S3, Figure S4). Using ImageJ, 1 pixel-wide line segments were traced along all dendrites and the portions of axons as stated below and previously described [18], [30]. Using DIC imaging and MAP2 staining or GFP fluorescence, the perimeter of the transfected soma was manually traced. Images displaying severe variability in focal plane were excluded from analysis. Regions where fasciculation or overlapping processes occurred were excluded from analysis.

We have observed that the axons originated directly from the soma in majority of the transfected neurons (Fig. 1, Figure S2B), while some neurons had their axons originated from their proximal dendrites (Figure S1, Figure S2B), consistent with the heterogeneity in the origin and location of the AIS [33]–[35]. Hence, the beginning of the axon was defined as the point where the axon originated from the soma or a proximal dendrite. The AIS was identified as an initial segment in the axon that was labeled with the AIS markers described above (Figure S2B). Our manual tracing along the axons revealed that the soma- or dendrite-derived AIS started at 4.4±2.6 µm and ended at 29.8±0.7 µm from the beginning of the axon in cultured hippocampal neurons (n = 8, ), consistent with the previous reports on the AIS length to be about 30 µm [33]–[35]. Thus, the background-subtracted mean fluorescence intensity of the soma, the axon within 0–30 µm of the beginning of the axon (AIS), the axon between 50–80 µm from the beginning of the axon (distal axon), and the major primary dendrites up to the disappearance of MAP2 staining (dendrite), were obtained for determination of the surface “AIS/Axon” and “Axon/Dendrite” ratios as previously described [18], [30] (Figure S2).

The color-merged images as well as inverted gray scale images were generated in Photoshop (Adobe Systems) as described [18]. Pseudo-color images were generated in ImageJ. For camera lucida drawings, the axon (identified by ankyrin-G or phospho IκBα Ser32 (14D4) staining of the AIS, or by the lack of MAP2 staining) from the inverted gray-scale image of the GFP-transfected neuron was traced using the neuronJ plugin for ImageJ as described [36] (Figure S3, Figure S4). Similar to the previously published camera lucida drawings of transfected hippocampal neurons [37], the cellular processes of the GFP-transfected neurons were colored in gray to increase the visibility of the axon, which was traced in black. For camera lucida drawings of the neurons that were not transfected with GFP, the cellular processes of the CD4- or HA-positive neurons were colored in gray (Figure S1, Figure S2).

### Immunoblot Analysis

At 5–9 days *in vitro*, the cultured hippocampal neurons were washed with ice-cold ACSF and harvested in ice-cold lysis buffer containing (in mM): 50 Tris, 150 NaCl, 2 EGTA, 1 EDTA, 1% Trition, 0.5% deoxycholic acid, 0.1% SDS (pH 7.4) supplemented with Halt protease inhibitors (Thermo Fisher Scientific) as described [38]. The resulting lysates were run on SDS-PAGE gels, transferred to a polyvinyl difluoride (PVDF) membrane (Immobilon, Millipore), and analyzed by immunoblotting as described [38]. Briefly, the membranes were blocked in 5% milk and 0.1% Tween-20 in Tris buffered saline for 1 hr, and then incubated with rabbit anti-CaM (1∶500 dilution) or anti-GAPDH antibody (1∶1000 dilution) in wash buffer (1% milk and 0.1% Tween-20 in tris buffered saline) overnight at 4°C. After incubating with horse radish peroxidase-conjugated secondary antibodies in wash buffer for 1 hr, the blots were treated with enhanced chemifluorescence substrate (ECL, Thermo Fisher Scientific), and developed with a Konica SRX-101A film processor.

### Immunoprecipitation

Immunoprecipitation was performed as described [22], [26], [39] with the following modification. HEK293T cells were plated on 60 mm cell culture dishes (BD Biosciences, 7×10^5^ cells per dish) maintained in Minimal Essential Medium containing 10% Fetal Calf Serum, 2 mM glutamine, 100 U/mL penicillin and 100 U/mL streptomycin at 37°C and 5% CO_2_. At 24 hr post plating, the cells were transfected with plasmids (total 1.7 µg) using FuGENE6 transfection reagent (Promega) according to manufacturer’s protocol. At 24 hr post transfection, the cells were washed with ice-cold PBS and solubilized in ice-cold immunoprecipitation (IP) buffer containing (in mM): 20 Tris-HCl, 100 NaCl, 2 EDTA, 5 EGTA, 1% Triton X-100 (pH 7.4) supplemented with Halt protease inhibitors (Thermo Fisher Scientific). The cells in IP buffer were incubated on ice for 15 min, followed by centrifugation at 17,000×g for 15 min at 4°C. The protein concentration of the resulting supernatant was determined using the BCA kit (Pierce). The lysate containing equal amount of proteins were incubated first with Protein A/G agarose beads (50 µL, Santa Cruz) for 1 hr 4°C. The pre-cleared supernatant was then incubated overnight at 4°C with Protein A/G-agarose beads (50 µL) and mouse anti-CD4 antibody (5 µg) or rabbit anti-KCNQ2 antibody (5 µg). After four washes with IP buffer, the immunoprecipitates were eluted with SDS sample buffer by incubating at 90°C for 5 min, and subjected to immunoblot analysis with anti-CD4 (1∶500 dilution), anti-CaM (1∶200 dilution), anti-KCNQ2 (1∶200 dilution), or anti-β-actin (1∶500 dilution).

### Electrophysiology

Coverslips containing dissociated rat hippocampal neurons were transfected, and 24–48 hr after transfection, the coverslips were transferred to the whole-cell patch clamp recording chamber in external solution containing (in mM): 126 NaCl, 3 KCl, 2 CaCl_2_, 2 MgSO_4_, 1 NaH_2_PO_4_, 25 NaHCO_3_ and 14 Dextrose, bubbled with 95% O_2_ and 5% CO_2_ (pH 7.4, 305–315 mOsm). The untransfected or GFP-positive pyramidal neurons were visually identified using an upright fluorescence microscope (Zeiss Axioscope) and the whole-cell patch clamp recordings were carried out immediately. All recordings were performed to obtain current clamp mode at room temperature (23–25°C) in the presence of the fast synaptic transmission blockers 6-cyano-7-nitroquinoxaline-2,3-dione (CNQX; 20 µM), DL-2-amino-5-phosphonopentanoic (DL-AP5; 100 µM) and bicuculline (20 µM) in external solution. Recording pipettes were pulled from glass capillaries with an outer diameter of 1.5 mm on a micropipette puller (P-97; Sutter Instruments), and had a resistance of 3–5 MΩ when filled with internal solution containing (in mM): 130 KMeSO_4_, 10 KCl, 10 HEPES/K-HEPES, 2 MgSO_4_, 0.5 EGTA and 3 ATP (pH 7.3, 285–295 mOsm). Current-clamp recordings were performed as previously described [40] with the following modifications. Neurons were held at –60 mV. Action potential firing rates (Hz) were measured upon delivering constant current pulses of 500 ms in the range 0 to 200 pA, and were averaged from 3 to 5 individual sweeps per current injection. Neurons were eliminated from further analysis if the access resistance changed by more than 20% over the recording period. Whole-cell recordings were made using a Multiclamp 700B amplifier (Molecular Devices). Recordings were filtered at 2 kHz and digitized at 10 kHz. Data was acquired and analyzed with a Digidata 1440A interface (Molecular Devices) and the pClamp suite of software (version 10.2; Molecular Devices). Recording analyses were performed using Clampfit software (version 10.2; Molecular Devices).

### Statistical Analyses

All fluorescence intensity and electrophysiology analyses were reported as mean ± SEM. ANOVA and post-ANOVA Tukey’s multiple comparison tests were performed to identify the statistically significant difference between groups of three or more, whereas the Student *t* test was performed for groups of two by Microsoft Excel with QI Macros 2013 plug-in. A priori value (p)<0.05 was considered statistically significant. The number of separate transfected cells for immunostaining and electrophysiology was reported as the sample size n.

## Results

### Fusion of KCNQ2 C-terminal tail preferentially targets CD4 to the axonal surface

To test whether CaM regulates preferential targeting of KCNQ channels to the axonal surface, we transfected rat dissociated hippocampal cultured neurons with the KCNQ3 subunit tagged with an extracellular hemagglutinin (HA) epitope (HA-KCNQ3) and KCNQ2 at 5–6 days *in vitro* (DIV) (Fig. 1A) when CaM is abundantly expressed (Fig. 1B). Since antibodies that recognize extracellular domains of endogenous KCNQ2 and KCNQ3 are not available, surface immunostaining of HA-KCNQ3 proteins has been used to demonstrate enrichment of HA-KCNQ3/KCNQ2 channels both at the AIS and more distally on axons [18]. The surface expression and function of HA-KCNQ3 has also been demonstrated in Xenopus oocytes [28]. The axon was identified as a MAP2-negative process from the transfected neuron (Fig. 1D, Figure S1). Surface immunostaining at 48 hr post-transfection revealed that HA-KCNQ3/KCNQ2 channels were preferentially expressed on the surface of axons originating directly from the soma compared to the somatodendritic surface (Fig. 1D). Consistent with the heterogeneity in the origin and location of the AIS [33]–[35], HA-KCNQ3/KCNQ2 channels were also enriched at the surface of axons originating from the proximal dendrites in some neurons (Figure S1). They were also highly concentrated at the surface of the AIS (Fig. 1D–E, Figure S1), which is consistent with previous reports of the distribution and function of endogenous KCNQ2 and KCNQ3 [5], [6], [10], [14], [41], [42].

The cluster of differentiation 4 (CD4), which is a non-neuronal single transmembrane glycoprotein, was used as a well-established trafficking reporter system [18], [30], [31] in which the C-terminal tail of KCNQ2 was fused to the C-termini of CD4 (CD4-Q2C) (Fig. 1C). Consistent with a previous report [18], we found that transfected CD4 proteins were uniformly distributed on the plasma membrane of hippocampal neurons whereas CD4-Q2C chimeric proteins were preferentially targeted to the axonal surface (Fig. 1F–G, Figure S2C). To quantify the polarized surface expression, we calculated the ratio of the mean surface fluorescence intensity of the distal axon to the major dendrites (Axon/Dendrite) [18], [30] and found that the surface “Axon/Dendrite” ratio was 0.5±0.1 for CD4, and 1.6±0.3 for CD4-Q2C (Figure S2E). Axonal enrichment of CD4-Q2C (Fig. 1F–G) closely mirrored the polarized axonal distribution of the intact HA-KCNQ3/KCNQ2 channels (Fig. 1D–E). Furthermore, the E810A and D812A mutations in KCNQ2 C-terminus, which disrupt ankyrin-G binding to KCNQ2 [6], [18], [20], reduced the surface “AIS/Axon” ratio of CD4-Q2C to 1, but had no effect on the surface “Axon/Dendrite” ratio (Figure S2). These results together suggest that preferential targeting of CD4-Q2C proteins to the axonal surface precedes their ankyrin-G-dependent enrichment at the AIS surface.

### Fusion of KCNQ2 C-terminal tail deficient in CaM binding fails to preferentially target CD4 to the axonal surface

A previous report [18] as well as our results (Fig. 1D–E, Figure S2) indicated that the membrane proximal region of the KCNQ2 C-terminal tail, upstream of the ankyrin-G binding domain, was sufficient to preferentially target CD4 to the axonal surface. This region of KCNQ2 contains helices A and B, which bind to CaM [22], [23] (Fig. 2A). To test whether CaM interaction with KCNQ2 regulates the targeting of CD4-Q2C to the axonal surface, we introduced point mutations in helix A of KCNQ2 including two BFNC mutations, L339R and R353G [43] (Fig. 2A). Mutations in the CaM-binding consensus IQ motif (L339R, I340E, and A343D) abolished co-immunoprecipitation of CD4-Q2C with CaM from HEK293T cell lysate (Fig. 2B), consistent with their ability to disrupt KCNQ2 interaction with CaM [24], [26].

**Figure 3:**
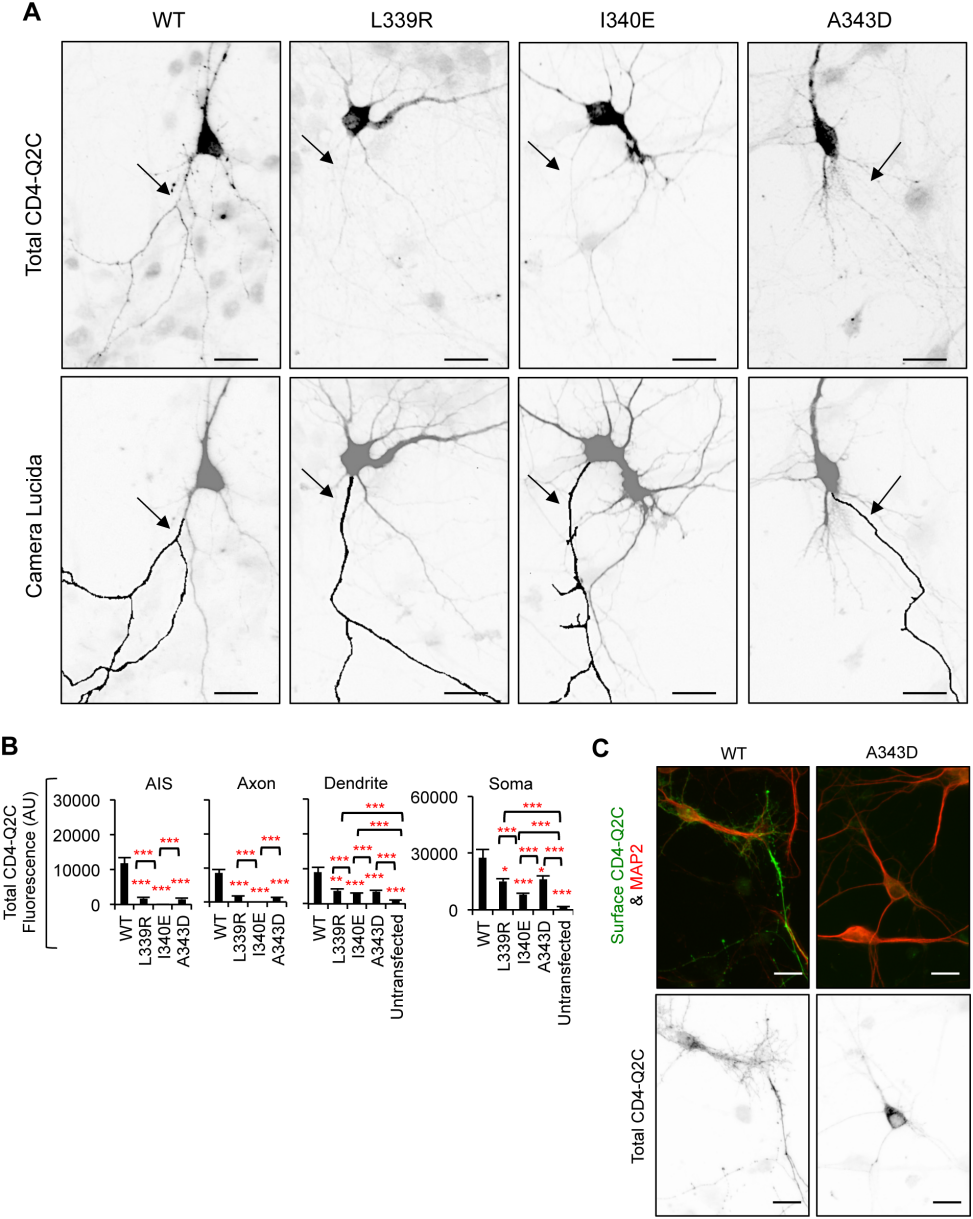
Mutations in IQ motif block CD4-Q2C expression in the axon. (A) Permeabilized immunostaining of CD4-Q2C wild-type (WT) or mutant (L339R, I340E, or A343D) in GFP-cotransfected hippocampal neurons (DIV 7–8). Camera lucida drawings (lower) of the inverted images of total CD4-Q2C (upper) show the soma and dendrites (gray) and an axon (black). Arrows indicate the AIS identified by phospho IκBα Ser32 (14D4) immunostaining. The L339R, I340E, and A343D mutations abolished total (surface and intracellular) expression of CD4-Q2C in the axon. (B) Background subtracted, mean intensity of total CD4 fluorescence in the AIS, distal axons, soma, and major dendrites. AU, arbitrary unit. The sample number for each construct used was as follows: WT (n = 18), L339R (n = 20), I340E (n = 12), A343D (n = 25), and untransfected (n = 20). Ave ± SEM (*p<0.05, **p<0.01, ***p<0.001). (C) Surface expression of A343D mutant CD4Q2C was absent (upper panel) although total (surface and intracellular) expression of A343D mutant CD4Q2C was evident in the MAP2-positive soma and proximal dendrites (lower panel). (A, C) Scale bars are 20 µm.

To determine the extent to which these mutations affect targeting of CD4-Q2C to the axonal surface, neurons were cotransfected with GFP, which allows visualization of all neurites. The axon was identified by immunostaining for the AIS marker phospho IκBα Ser32 (14D4) (Fig. 2, Figure S3). Surface immunostaining revealed that mutations in the CaM-binding consensus IQ motif (L339R, I340E, and A343D) decreased surface expression of CD4-Q2C at the AIS and distal axons to background fluorescence intensities of untransfected neuronal dendrites (Fig. 2C, E) and reduced the surface “Axon/Dendrite” ratio to nearly 0 (Fig. 2D). The surface “AIS/Axon” ratios for these mutants were not calculated because the majority of these mutants had background-subtracted mean fluorescence values of 0 at the AIS and distal axon. Furthermore, these mutant proteins were intracellularly excluded from the AIS and distal axons (Fig. 3A–C) and displayed reduced somatodendritic expression (Fig. 3B).

**Figure 4:**
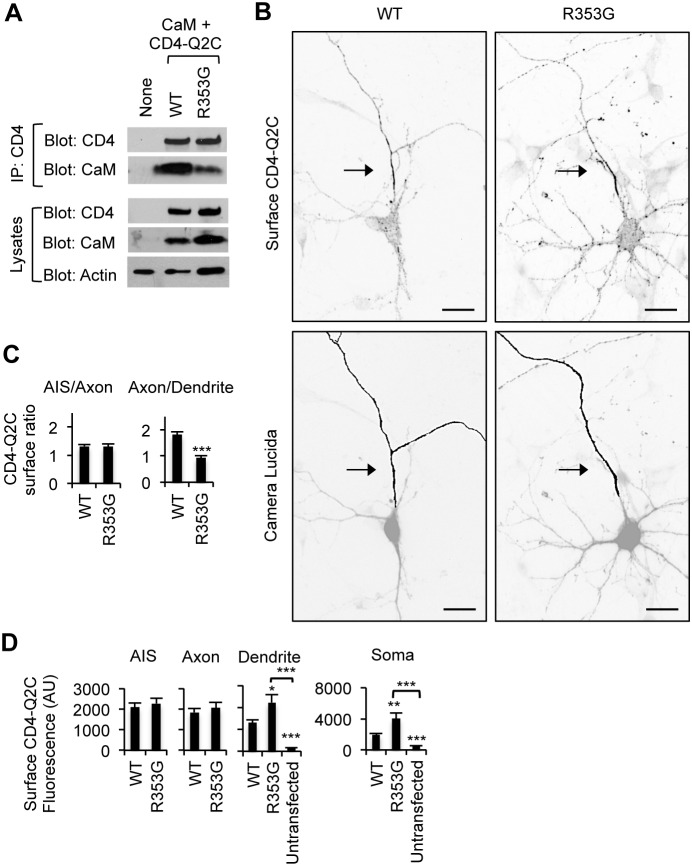
The BFNC R353G mutation blocks axonal enrichment of surface CD4-Q2C. (A) The R353G mutation reduced but did not abolish co-immunoprecipitation of CaM with CD4-Q2C from transfected HEK293T cells. β-actin served as a loading control for total cell lysates (B) Surface immunostaining of WT or R353G mutant CD4-Q2C in hippocampal neurons (DIV 7–8). Camera lucida drawings (lower) of the inverted images of surface CD4-Q2C (upper) show the soma and dendrites (gray) and an axon (black). The axon was identified by the lack of MAP2 immunostaining in the neurons cotransfected with GFP. The R353G mutation blocked enrichment of CD4-Q2C on the axonal surface by increasing its somatodendritic surface expression. Arrows mark the main axon. Scale bars are 20 µm. (C) In comparison to WT, the surface “Axon/Dendrite” fluorescence ratio of CD4-Q2C was reduced to 1 by the R353G mutation, whereas the surface “AIS/Axon” ratio was unaffected. (D) Background subtracted, mean intensity of surface CD4 fluorescence in the AIS, distal axons, soma, and major dendrites. The R353G mutation increased CD4-Q2C expression at the somatodendritic surface compared to WT. The sample number for each construct used in (C, D) was as follows: WT (n = 23), R353G (n = 18), and untransfected (n = 15). AU, arbitrary unit. Ave ± SEM (*p<0.05, **p<0.01, ***p<0.001).

**Figure 5:**
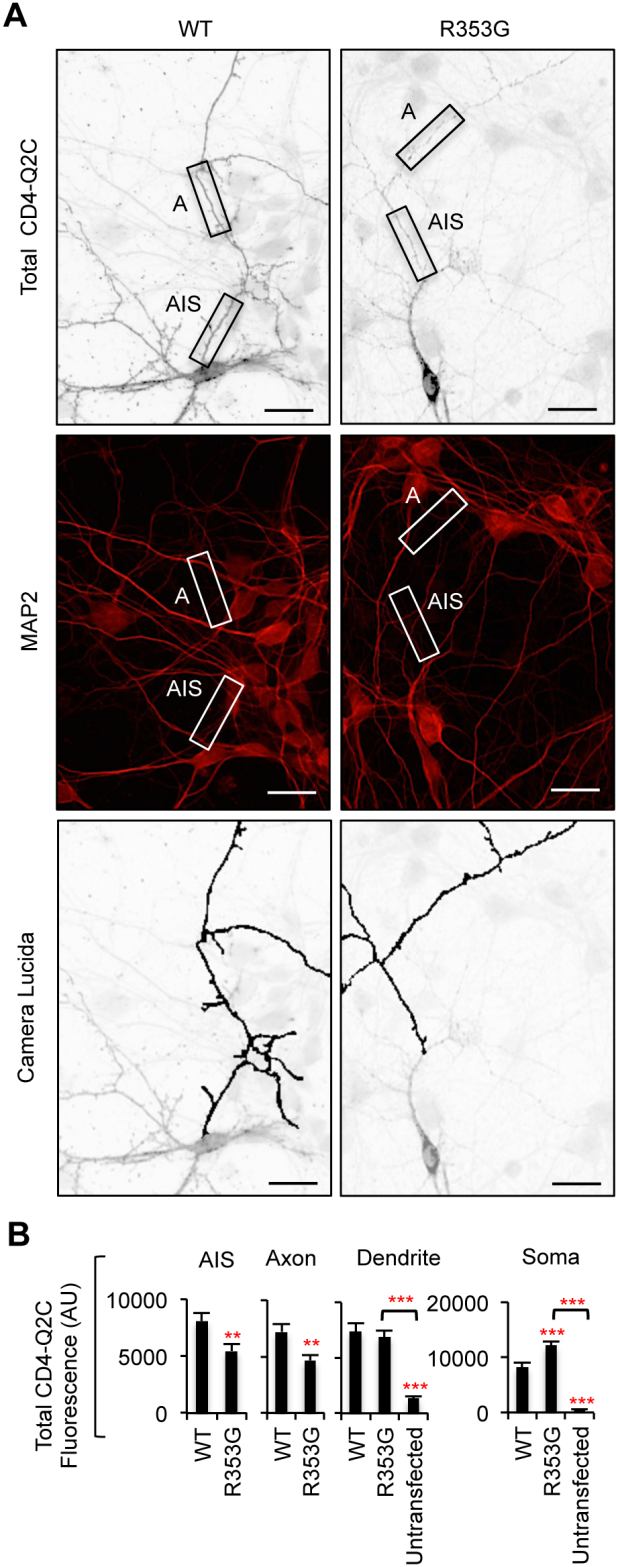
The R353G mutation reduces CD4-Q2C expression in the axon. (A) Permeabilized immunostaining of wild-type (WT) or R353G mutant CD4-Q2C in hippocampal neurons (DIV 7–8) cotransfected with GFP. Neuronal soma and dendrites were identified by MAP2 immunostaining (middle). The insets in the representative inverted images of total CD4-Q2C show the initial segment (AIS) and distal segment (A) of the MAP2-negative axon in transfected neurons. Camera lucida drawings (lower) of the neuronal images (upper) show the soma and dendrites (gray) and an axon (black). The R353G mutation reduced but did not abolish total (surface and intracellular) expression of CD4-Q2C from the axon. Scale bars are 20 µm. (B) Background subtracted, mean intensity of total CD4 fluorescence in the AIS, distal axons, soma, and major dendrites. AU, arbitrary unit. The sample number for each construct used was as follows: WT (n = 23), R353G (n = 18), and untransfected (n = 15). AU, arbitrary unit. Ave ± SEM (**p<0.01, ***p<0.001).

The R353G mutation located distal to the IQ motif (Fig. 2A) has been shown to moderately reduce CaM binding to KCNQ2 in HEK293T cells [24], [26]. Consistent with previous reports, the same mutation decreased but did not abolish co-immunoprecipitation of CD4-Q2C with CaM (Fig. 4A). In contrast to mutations in the IQ motif (Fig. 2–3), the R353G mutation increased somatodendritic surface expression of CD4-Q2C without affecting axonal surface expression (Fig. 4B, D), thereby decreasing the surface “Axon/Dendrite” ratio to 1 (Fig. 4C). The surface CD4-Q2C R353G proteins also displayed a punctate distribution in the soma and dendrites compared to the CD4-Q2C WT (Fig. 4B). The surface “AIS/Axon” ratio was unaffected by the R353G mutation compared to wild-type CD4-Q2C (Fig. 4C). Permeabilized immunostaining revealed that total (surface and intracellular) expression of the R353G mutant CD4-Q2C proteins increased in the soma but modestly decreased in the axons (Fig. 5A–B). These results together indicate that fusion of KCNQ2 C-terminal tail deficient in CaM binding fails to preferentially target CD4 to the axonal surface.

### Mutations that block CaM binding impair trafficking of CD4-Q2C from the ER to the axon

**Figure 6:**
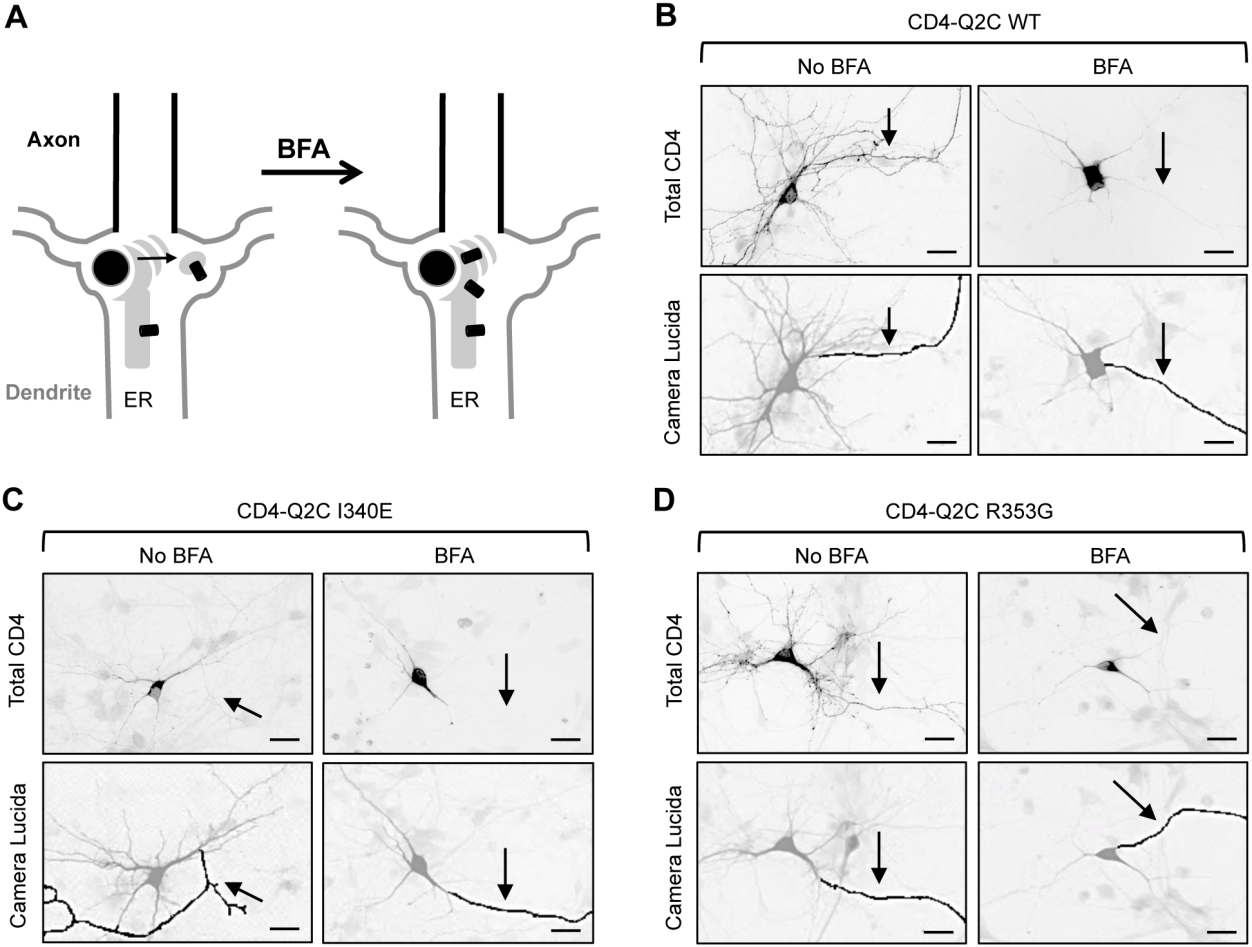
CD4-Q2C accumulates in the ER after BFA treatment. (A) Schematic drawings of a hippocampal neuron containing a continuous network of the ER from the soma to the dendrites. Treatment with brefeldin-A (BFA) leads to inhibition of anterograde transport from the ER to the Golgi complex [44]. (B–C) The cultured hippocampal neurons (DIV 5) were treated with vehicle control (No BFA) or BFA (0.75 µg/ml) at 30 min post transfection with GFP and CD4-Q2C wild-type (WT) (B), I340E mutant (C), or R353G mutant (D). At 16 hr post-BFA treatment, permeabilized immunostaining was performed to visualize total (surface and intracellular) expression of CD4-Q2C (inverted images, upper). BFA treatment caused newly synthesized wild-type and all mutant proteins to accumulate at perinuclear regions in the soma and proximal dendrites. Arrows mark the main axon identified by the lack of MAP2 immunostaining in the neurons cotransfected with GFP. Camera lucida drawings (lower) show the soma and dendrites (gray) and an axon (black). Scale bars are 20 µm.

KCNQ2 subunits deficient in CaM binding are retained in the ER of HEK293T cells [24], [26], suggesting the possibility that preferential targeting of CD4-Q2C proteins to the axonal surface may involve their CaM-dependent trafficking from the ER to the axon in hippocampal neurons. To test this hypothesis, we treated the transfected neurons at 30 min post-transfection with brefeldin-A (BFA), which is a reversible inhibitor of anterograde transport from the ER to the Golgi complex [44] (Fig. 6A). Permeabilized immunostaining of transfected neurons treated with vehicle control for 16 hr revealed that total (surface and intracellular) expression of wild-type and R353G mutant CD4-Q2C proteins were found throughout the neuron, whereas expression of I340E mutant proteins were confined to the perinuclear region of the soma and proximal dendrites (Fig. 6B–D). In contrast, BFA treatment for 16 hr caused newly synthesized wild-type and all mutant proteins to accumulate at perinuclear regions in the soma and proximal dendrites (Fig. 6B–D). Wild-type and R353G mutant proteins but not I340E mutant proteins were also found in the distal dendrite of some transfected neurons at 16 hr post-BFA treatment (Fig. 6B–D). Since rough ER is prominent in the neuronal soma and proximal dendrites whereas smooth ER predominates in the distal dendrites and dendritic spines [45]–[47], these results suggest that newly synthesized CD4-Q2C proteins are retained mostly in the rough ER after BFA treatment.

**Figure 7:**
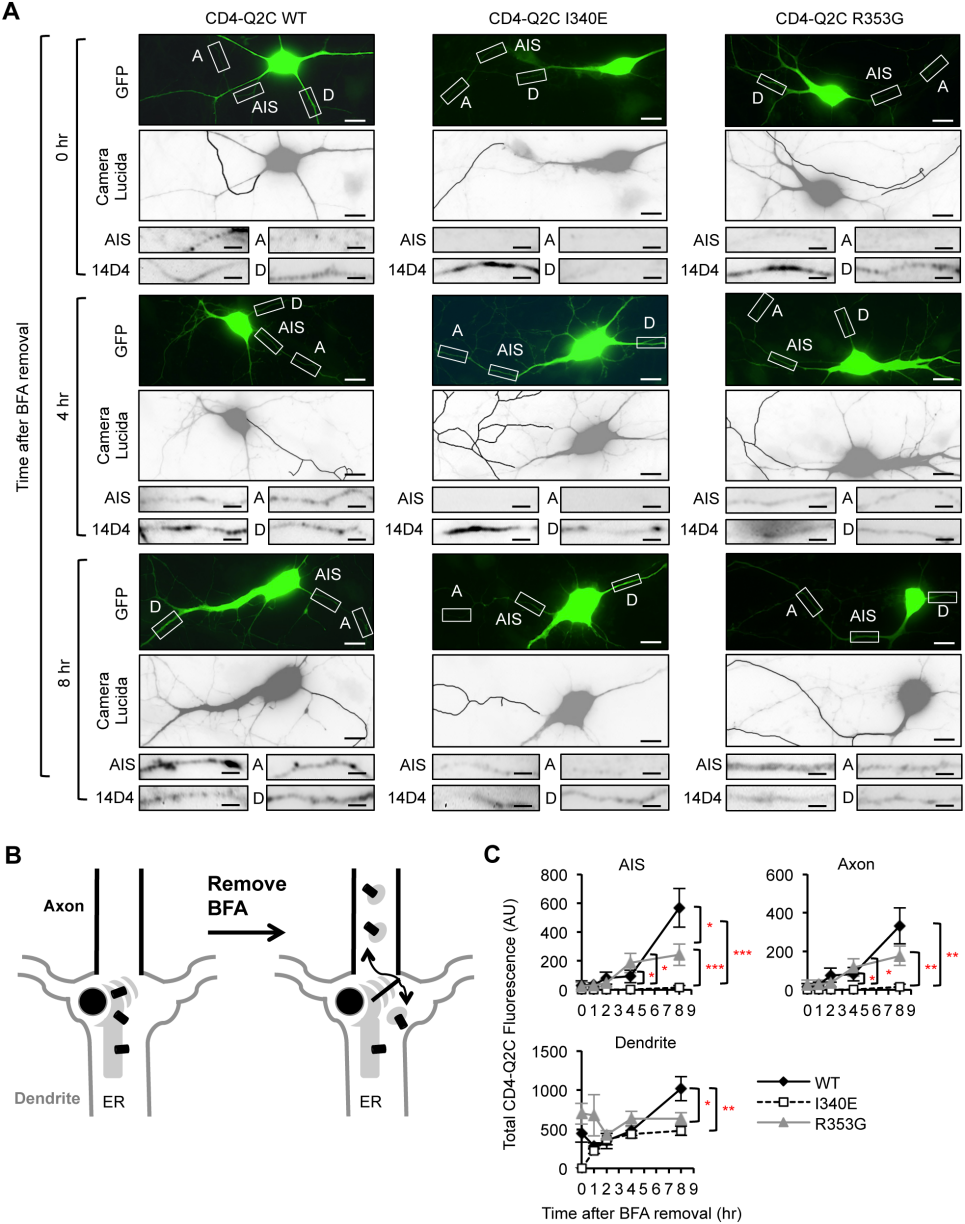
The I340E mutation impairs CD4-Q2C trafficking from the ER to the axon. Pulse-chase assay of wild-type (WT) or mutant (I340E and R353G) CD4-Q2C proteins after BFA washout. At 16 hr post BFA treatment, BFA was washed out and the neurons were placed in the cell culture incubator for 0, 1, 2, 4, and 8 hr. Permeabilized immunostaining was performed to visualize total (surface and intracellular) expression of CD4-Q2C in neurons cotransfected with GFP. The axon was identified by immunostaining with the AIS marker phospho IκBα Ser32 (14D4). (A) Representative images of CD4-Q2C WT or mutant proteins at 0, 4, and 8 hr after BFA washout. Camera lucida drawings (middle) of the GFP-transfected neurons (upper) show the soma and dendrites (gray) and an axon (black). Scale bars in the upper and middle panels are 10 µm. The small lower panels are representative inverted images of CD4-Q2C in the AIS (AIS), distal axons (A), and dendrites (D) in transfected neurons (insets). Scale bars of the small lower panels are 5 µm. (B) Schematic drawings of a hippocampal neuron containing CD4-Q2C after BFA washout. (C) Background subtracted, mean intensity of total CD4 fluorescence in the AIS, distal axons, and major dendrites. The wild-type CD4-Q2C proteins gradually appeared at the AIS and distal axons upon BFA washout. The I340E mutation abolished whereas the R353G mutation reduced the accumulation of CD4-Q2C at the AIS and distal axons. The sample numbers per time point for each construct (n = 8–21). AU, arbitrary unit. Ave ± SEM (*p<0.05, **p<0.01, ***p<0.001).

BFA was then removed and the neurons were fixed at various time points to “chase” the trafficking of CD4-Q2C proteins from the ER by permeabilized immunostaining (Fig. 7A–B). The wild-type CD4-Q2C proteins gradually appeared in a diffused distribution as well as in punctate structures in the AIS and distal axons upon BFA removal (Fig. 7A, C). In contrast, I340E mutant proteins were mostly absent from the AIS and distal axon for the duration of the 8 hr BFA washout (Fig. 7A, C), consistent with our previous results for the I340E mutation, which blocked both surface and intracellular expression of CD4-Q2C throughout the axon (Fig. 2–3, 6C). In contrast, the R353G mutant proteins were found in the AIS and distal axon, albeit at reduced levels compared to wild-type CD4-Q2C at 8 hr post-BFA washout (Fig. 7A, C). BFA removal also caused wild-type and all mutant proteins to distribute diffusely in the dendrites with occasional accumulation in punctate structures at time points earlier than in the axons (Fig. 7A, C). Interestingly, the expression of I340E mutant CD4-Q2C was initially reduced in the dendrites compared to wild-type and R353G mutant proteins (Fig. 7A, C). However, the level of R353G mutant proteins at 8 hr post BFA washout was similar to the amount of I340E mutant protein in dendrites, which was approximately half the amount of wild-type CD4-Q2C (Fig. 7A, C). These results together suggest that disruption of CaM binding to KCNQ2 impairs the trafficking of CD4-Q2C proteins from the ER to the AIS and distal axons.

### Disruption of CaM binding to KCNQ2 inhibits enrichment of HA-KCNQ3/KCNQ2 channels at the axonal surface

**Figure 8:**
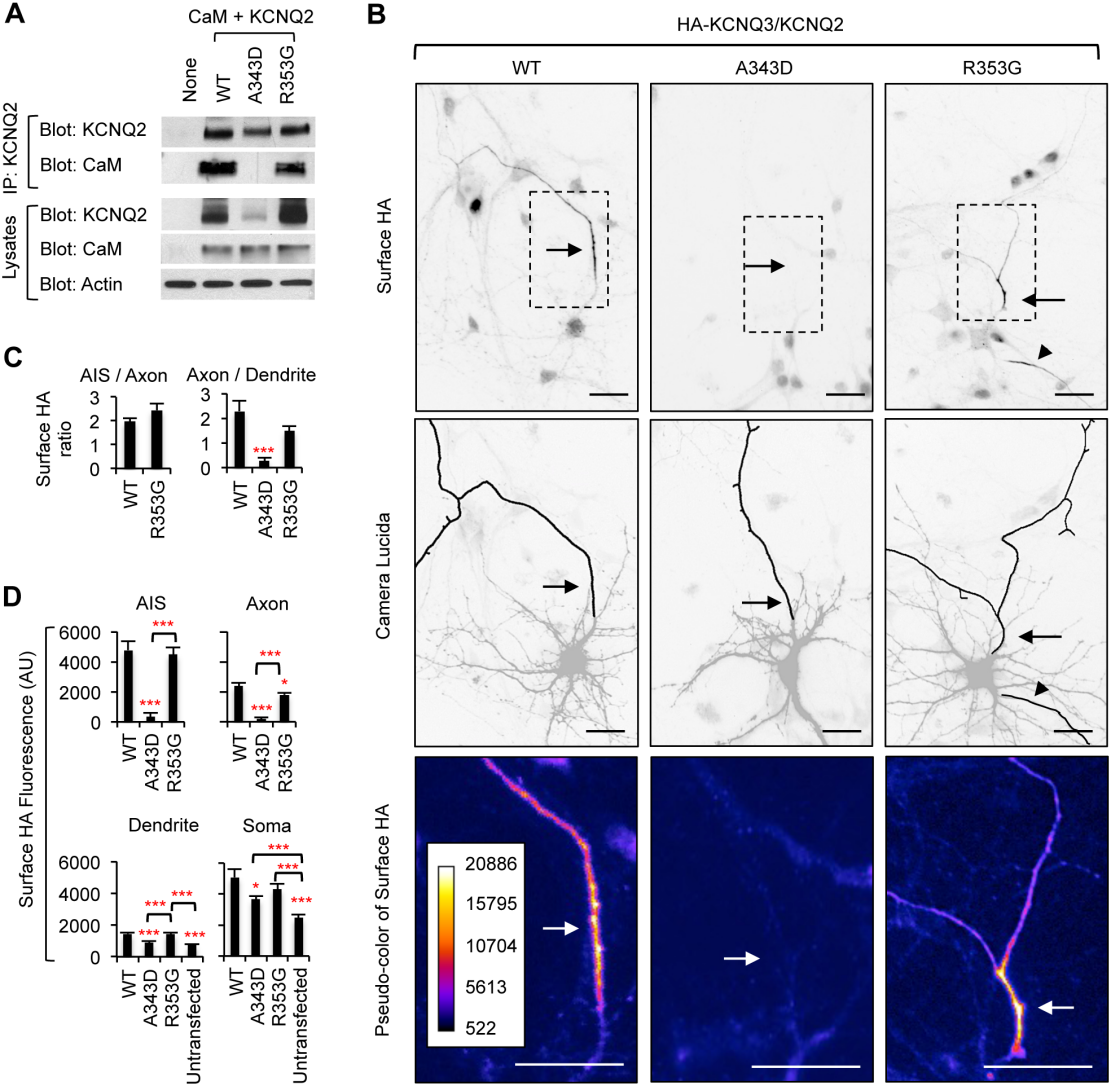
The A343D mutation blocks axonal enrichment of surface HA-KCNQ3/KCNQ2 channels. (A) Lysates from HEK293T cells expressing CaM and wild-type KCNQ2 (WT) or mutant KCNQ2 (A343D and R353G) were subjected to immunoprecipitation (IP) with the KCNQ2 antibody. Immunoprecipitation and total cell lysates were analyzed by immunoblotting for KCNQ2 and CaM. β-actin served as a loading control. The A343D mutation abolished whereas the R353G modestly reduced co-immunoprecipitation of CaM with KCNQ2. (B) Representative inverted images of surface HA-KCNQ3 in hippocampal neurons cotransfected with GFP and KCNQ2 WT or mutants (A343D and R353G). The A343D but not the R353G mutation abolished surface expression of HA-KCNQ3/KCNQ2 at the axon, which was identified by immunostaining for the AIS marker, phospho IκBα Ser32 (14D4) (Figure S4). Camera lucida drawings (middle) of neuronal images (upper) show the soma and dendrites (gray) and an axon (black). Pseudo-color images (lower) of the insets in the neuronal images (upper) display differences in the surface HA intensity. Arrows indicate the AIS. Arrowheads mark another axon. Scale bars: 20 µm. (C) The surface “Axon/Dendrite” ratio was reduced by the A343D but not the R353G mutation. The surface AIS/distal axon ratio for A343D mutant channels was not calculated due to their absence at the axonal and AIS surface. (D) Background subtracted, mean intensity of surface HA fluorescence in the AIS, distal axons, soma, and major dendrites. The sample number for each construct was as follows: WT (n = 27), A343D (n = 22), R353G (n = 21), and untransfected (n = 15). AU, arbitrary unit. Ave ± SEM (*p<0.05, **p<0.01, ***p<0.001).

Our results with CD4-Q2C proteins (Fig. 2–7) suggest that enrichment of intact KCNQ channels at the axonal surface may be mediated by CaM interaction with the IQ motif of KCNQ2. To test this hypothesis, we first examined the effect of A343D and R353G mutations on the interaction between CaM and intact KCNQ2 (Fig. 8A). Despite transfecting the same amount of each expression plasmid in HEK293T cells, we found that expression of the A343D mutant KCNQ2 subunits was consistently lower compared to wild type or the R353G mutant KCNQ2 subunits (Fig. 8A). Nonetheless, the immunoprecipitated amount of wild type and mutant KCNQ2 subunits using the same quantity of anti-KCNQ2 antibodies were nearly equal (Fig. 8A). This is most likely because the amount of antibodies used for immunoprecipitation was far less sufficient to immunoprecipitate all of transfected KCNQ2 proteins from the HEK lysate due to high level of expression of KCNQ2 proteins including A343D mutant proteins. Importantly, the A343D but not the R353G mutation abolished co-immunoprecipitation of KCNQ2 with CaM from HEK293T cell lysate (Fig. 8A).

**Figure 9:**
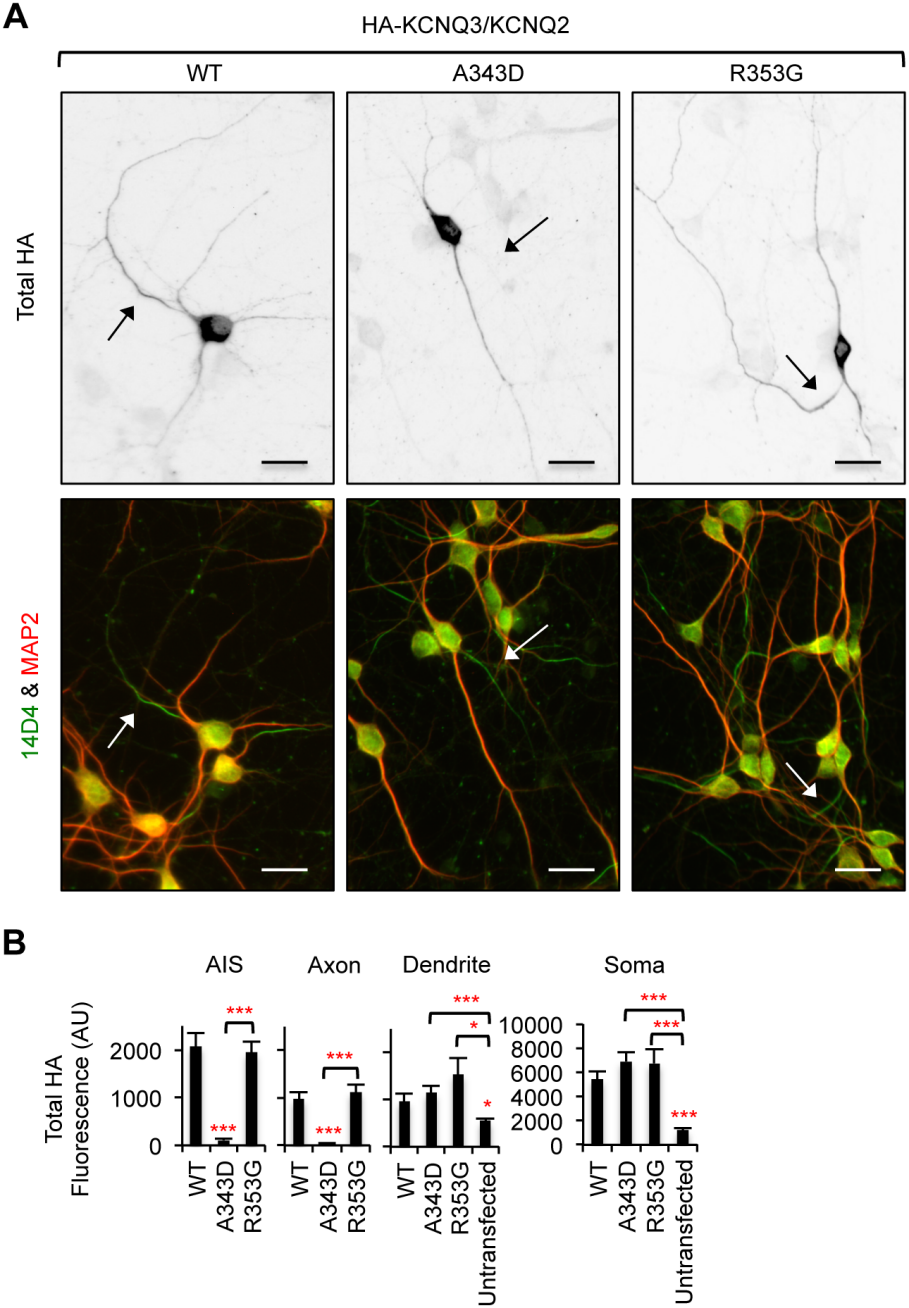
The A343D mutation abolishes axonal expression of HA-KCNQ3/KCNQ2 channels. (A) Permeabilized immunostaining was performed to visualize total (surface and intracellular) expression of HA-KCNQ3/KCNQ2 WT or mutant (A343D and R353G) channels (inverted images, upper). The axon was identified by the AIS marker phospho IκBα Ser32 (14D4) whereas neuronal soma and dendrites were visualized by MAP2 immunostaining (lower). The A343D but not the R353G mutation abolished total expression of HA-KCNQ3/KCNQ2 channels at the axon. Arrows indicate the AIS. Scale bars are 20 µm. (B) Background subtracted, mean intensity of total HA fluorescence in the AIS, distal axons, soma, and major dendrites. The sample number for each construct was as follows: WT (n = 27), A343D (n = 22), R353G (n = 21), and untransfected (n = 15). AU, arbitrary unit. Ave ± SEM (*p<0.05, ***p<0.001).

Next we performed surface immunostaining in hippocampal neurons transfected with GFP, HA-KCNQ3, and wild-type KCNQ2 or mutant KCNQ2 (A343D and R353G). Robust surface expression of wild-type HA-KCNQ3/KCNQ2 channels was detected at the AIS and distal axons compared to dendrites (Fig. 8B, Figure S4) [18]. In contrast, the A343D mutation, which blocked CaM binding to KCNQ2 (Fig. 8A) [24], [26], abolished surface and intracellular expression of HA-KCNQ3/KCNQ2 channels at the AIS and distal axons (Fig. 8B, D, 9A–B) and reduced the surface “Axon/Dendrite” ratio to below 1 (Fig. 8C). The A343D mutation also decreased surface expression in the soma and dendrites compared to the wild type (Fig. 8B, D). Despite a modest reduction of CaM binding to KCNQ2 by the R353G mutation (Fig. 8A) [24], [26], the R353G mutant channels were localized to the AIS and axonal surface to a similar extent as the wild-type channels (Fig. 8B–D). Although the R353G mutation increased somatodendritic surface expression of CD4-Q2C (Fig. 4), the same mutation in full-length KCNQ2 had no effect on the surface and intracellular expression of HA-KCNQ3/KCNQ2 channels throughout the neuron compared to the wild type (Fig. 8B, D, 9A, B). These results indicate that enrichment of HA-KCNQ3/KCNQ2 channels at the axonal surface was impaired by the A343D mutation in IQ motif that blocks CaM binding.

### Disruption of CaM binding to KCNQ2 impairs trafficking of HA-KCNQ3/KCNQ2 channels from the ER to the axon

**Figure 10:**
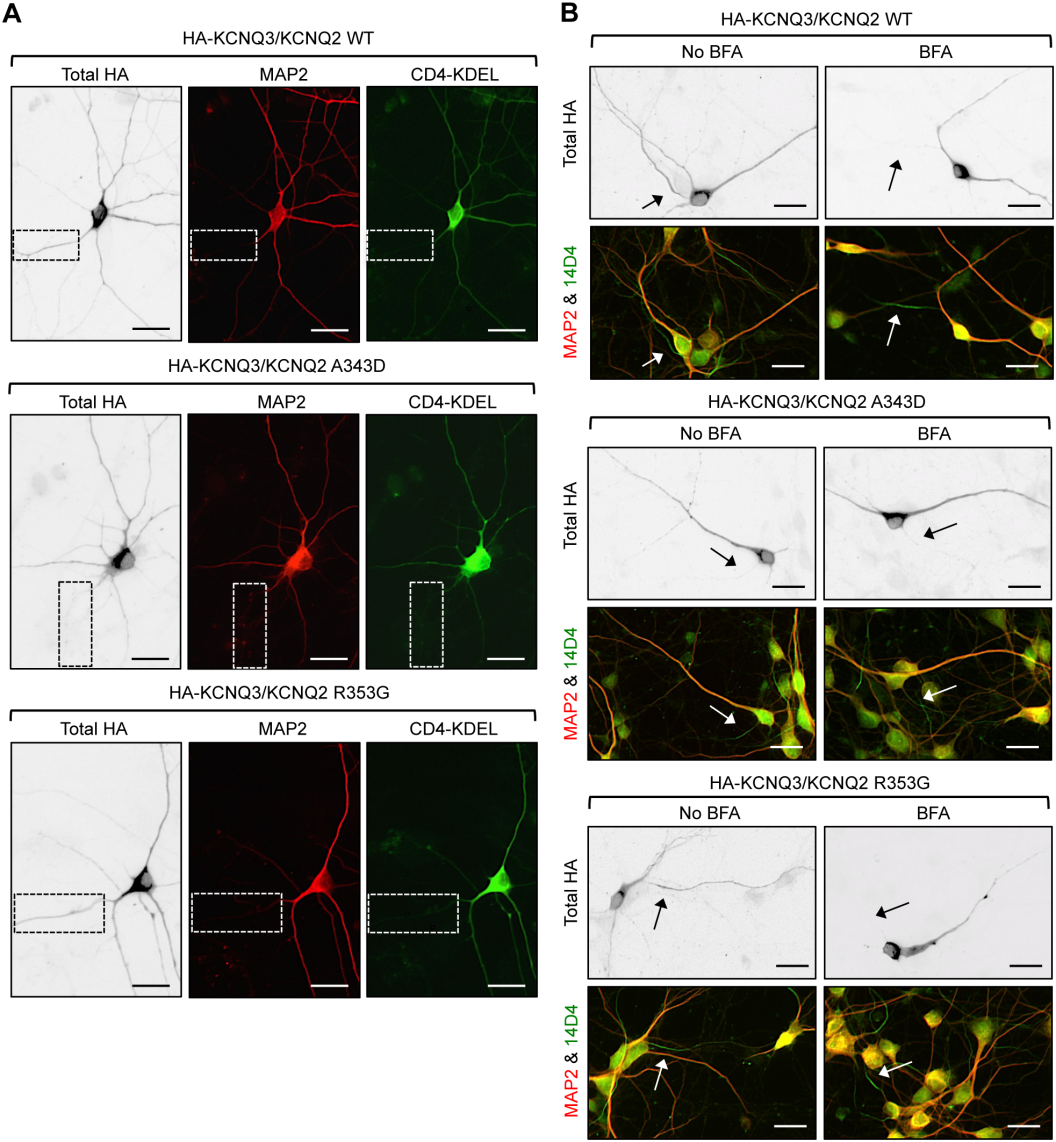
The HA-KCNQ3/KCNQ2-A343D mutant channels are absent from the ER-negative axon. (A) Permeabilized immunostaining of HA-KCNQ3/KCNQ2 channels (inverted images, left) and MAP2 (middle) were performed in cultured hippocampal neurons (DIV 7) cotransfected with the CD4 proteins harboring the ER retention/retrieval motif (CD4-KDEL, green). The wild type (WT) and R353G mutant channels were found at the axons where the A343D mutant channels and CD4-KDEL proteins were absent. The insets show the major axon. (B) The cultured hippocampal neurons (DIV 5) were treated with vehicle control (No BFA) or BFA (0.75 µg/ml) at 30 min post transfection with HA-KCNQ3 and KCNQ2 wild-type (WT), or mutant (A343D and R353G). At 16 hr post-BFA treatment, permeabilized immunostaining was performed to visualize total (surface and intracellular) expression of HA-KCNQ3/KCNQ2 channels. The axon was identified by the AIS marker phospho IκBα Ser32 (14D4), whereas neuronal soma and dendrites were visualized by MAP2 immunostaining. BFA treatment caused newly synthesized wild-type and all mutant channels to accumulate at perinuclear regions in the soma and dendrites but not axons. Arrows mark the AIS. Scale bars are 20 µm.

To examine whether the A343D mutation enhances the retention of HA-KCNQ3/KCNQ2 channels in the ER, we first examined their colocalization with the CD4 proteins harboring a KDEL ER retention signal (CD4-KDEL) [29], . The CD4-KDEL proteins were concentrated at the perinuclear region of the soma and displayed diffused distribution in the proximal and distal dendrites (Fig. 10A), consistent with the continuous network of the ER from the soma to the dendrites in hippocampal neurons [45], [47]. Wild type and all mutant HA-KCNQ3/KCNQ2 channels displayed significant colocalization with CD4-KDEL proteins in the soma and dendrites (Fig. 10A), suggesting that majority of the channels reside in rough and smooth ER of hippocampal cultured neurons. The wild type and R353G mutant channels were also found at the AIS and distal axons, regions where the A343D mutant channels and the CD4-KDEL proteins were absent (Fig. 10A), indicating that the A343D mutation in the IQ motif of KCNQ2 enhances the localization of HA-KCNQ3/KCNQ2 channels in the ER.

To test whether CaM binding to KCNQ2 promotes trafficking of HA-KCNQ3/KCNQ2 channels from the ER to the axon, we performed pulse-chase experiments after removal of BFA. At 16 hr treatment with vehicle control, the wild-type and R353G mutant HA-KCNQ3/KCNQ2 channels were expressed throughout the neuron including axons whereas the A343D mutant channels were found in the soma and dendrites but not axons (Fig. 10B), consistent with the total expression of these channels (Fig. 9, 10A). At 16 hr post BFA treatment, newly synthesized wild-type and mutant channels accumulated in the soma and proximal dendrites (Fig. 10B, Figure S5). Although they were found in distal dendrites, they were absent from the distal axons after BFA treatment (Fig. 10B, Figure S5).

**Figure 11:**
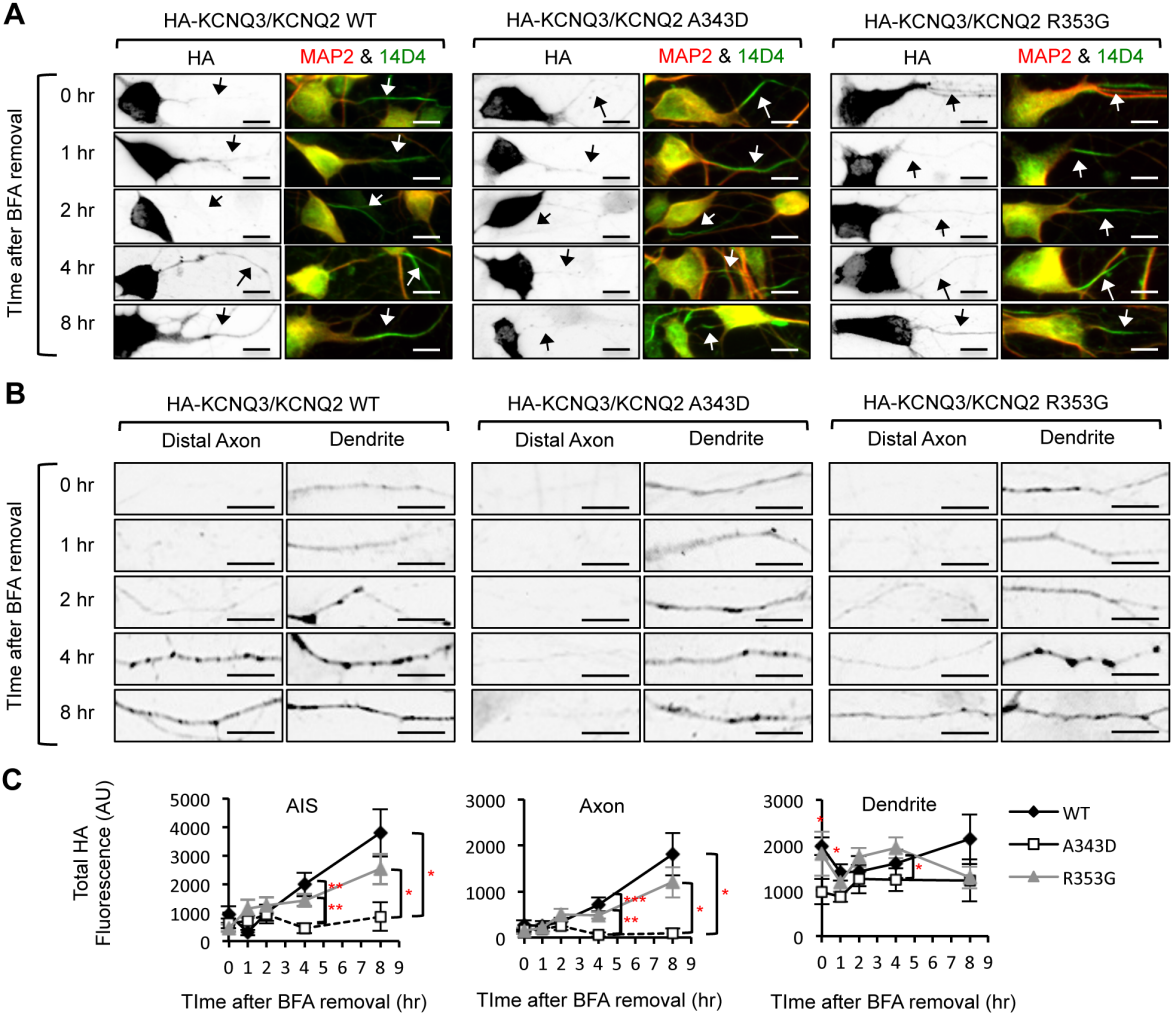
The A343D mutation impairs HA-KCNQ3/KCNQ2 trafficking from the ER to the axon. (A–B) Pulse-chase assay of wild-type (WT) or mutant (A343D and R353G) HA-KCNQ3/KCNQ2 channels after BFA washout. Permeabilized immunostaining was performed to visualize total (surface and intracellular) expression of HA-KCNQ3/KCNQ2 channels in the soma and AIS (A) as well as in the distal axons and dendrites (B) at indicated time points post-BFA removal (inverted images). The axon was identified by the AIS marker phospho IκBα Ser32 (14D4), whereas neuronal soma and dendrites were visualized by MAP2 immunostaining. Arrows indicate the AIS. Scale bars: 10 µm. (C) Background subtracted, mean intensity of total HA fluorescence in the AIS, distal axon, and major dendrites. Upon BFA removal, the A343D mutation but not the R353G mutation markedly reduced the appearance of HA-KCNQ3/KCNQ2 at the AIS and distal axon for the duration of the 8 hr BFA washout. The sample numbers were (n = 8–22) per time point for each construct. AU, arbitrary unit. Ave ± SEM (*p<0.05, **p<0.01, ***p<0.001).

Upon BFA removal, the wild-type HA-KCNQ3/KCNQ2 channels and the R353G mutant channels gradually appeared in a diffused distribution as well as in punctate structures in the AIS and distal axons for the duration of the 8 hr BFA washout (Fig. 11A–C, Figure S5). In contrast, the A343D mutant channels displayed minimal expression at the AIS and distal axons for the duration of the 8 hr BFA washout (Fig. 11A–C, Figure S5). Wild-type and mutant HA-KCNQ3/KCNQ2 channels were also found at the distal dendrites for the duration of the 8 hr BFA washout (Fig. 11B–C, Figure S5), consistent with their steady-state localization in the ER (Fig. 10A). Although the expression of the R353G mutant channels appeared slightly elevated at the dendrites compared to the A343D mutant channels at 4 hr post BFA washout, the average dendritic expression of the wild-type channels was not statistically different from that of the R353G and A343D mutant channels by 8 hr post BFA washout (Fig. 11B–C). These results together indicate that disruption of CaM interaction with KCNQ2 by the A343D mutation impairs the trafficking of HA-KCNQ3/KCNQ2 channels from the ER to the axon.

### Hippocampal neuronal excitability decreases upon expression of wild-type KCNQ2 but not mutant KCNQ2 deficient in CaM binding

**Figure 12:**
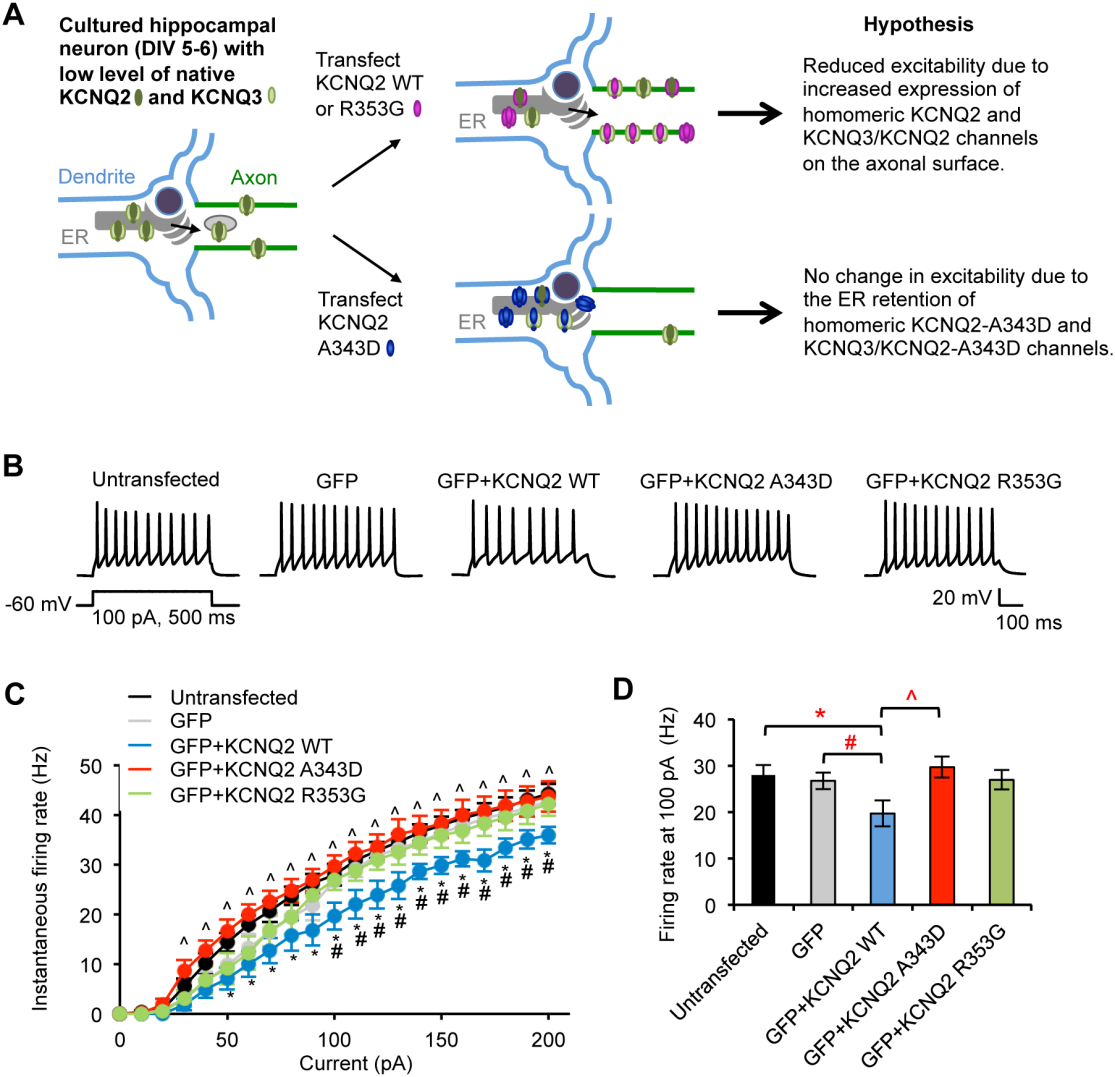
Expression of wild-type KCNQ2 but not KCNQ2-A343D or KCNQ2-R353G decreases neuronal excitability. (A) Hypothesis by which exogeneously expressed KCNQ2 subunits affect neuronal excitability. At an early stage of hippocampal culture, the level of endogenous KCNQ2/KCNQ3 channels is low [42]. Transfection of wild-type KCNQ2 or R353G mutant KCNQ2 would reduce action potential firing by increasing axonal surface expression of KCNQ2-containing homomeric channels and heteromeric channels formed with endogenous KCNQ3 subunits. In contrast, expression of A343D mutant KCNQ2 subunits deficient in CaM binding would have little effect on action potential firing due to their inability to exit the ER and express on the axonal surface. (B–D) Whole-cell patch clamp recording in cultured hippocampal neurons (DIV 6–8) that were cotransfected with GFP and KCNQ2 WT or mutants (A343D and R353G). Spike trains were evoked in untransfected or GFP-positive pyramidal neurons by delivering constant somatic current pulses for 500 ms duration at a resting potential of –60 mV. (B) Representative traces of action potentials induced by 100 pA injection. (C) Average instantaneous AP firing rates (Hz) induced by 0–200 pA injection into untransfected neurons (n = 10), or neurons transfected with GFP (n = 10), GFP + KCNQ2 WT (n = 10), GFP + KCNQ2 A343D (n = 10), or GFP + KCNQ2 R353G (n = 9). (D) Summary plots illustrating the effect of transfection on instantaneous firing rates at 100 pA. Ave ± SEM (*p<0.05 for untransfected vs. WT, #p<0.05 for GFP vs. WT, ∧p<0.05 for WT vs. A343D).

We next investigated the physiological relevance of CaM-dependent axonal enrichment of KCNQ channels. Previous studies using whole-cell patch clamp recording of action potentials have demonstrated that axonal rather than somatic KCNQ channels suppress hippocampal neuronal excitability [14], [17]. Furthermore, we showed that the wild-type KCNQ2 but not the A343D mutant KCNQ2 enriched HA-KCNQ3 at the axonal surface in hippocampal neurons cultured at 7–8 DIV (Fig. 8). Since expression of exogenous KCNQ2 has been previously shown to increase KCNQ current in hippocampal neurons cultured at 10 DIV when a relatively low level of native KCNQ2/3 channels are present [42], we hypothesized that expression of wild-type KCNQ2 subunits would reduce action potential firing by increasing axonal surface expression of both homomeric KCNQ2 channels and heteromeric channels formed with endogenous KCNQ3 subunits (Fig. 12A). In addition, we further hypothesized that expression of KCNQ2-A343D subunits deficient in CaM binding would have little effect on action potential firing, owing to their inability to exit the ER and express on the axonal surface (Fig. 12A).

To test this hypothesis, we performed whole-cell patch clamp recording of action potentials as described [40] in cultured hippocampal neurons (6–8 DIV). Transient expression of wild-type KCNQ2 together with GFP decreased action potential firing rates compared to untransfected neurons for all current injections from 50 pA and neurons expressing GFP for all current injections from 100 pA (Fig. 12B–C). These results suggest that the transfected wild-type KCNQ2 subunits form functional homomeric channels and/or heteromeric channels with endogenous KCNQ3 subunits, yielding outward K^+^ current in transfected neurons. The neurons expressing wild-type KCNQ2 displayed a 26–30% decrease in the mean firing frequency induced by 100 pA current injection (19.7±2.8 Hz, p<0.05) compared to untransfected neurons (28.0±2.2 Hz) and GFP-transfected neurons (26.7±1.8 Hz) (Fig. 12D), which is consistent with wild-type KCNQ2 exerting its effect in the axon rather than in the soma [14], [17].

In contrast to the expression of wild-type KCNQ2 which reduced action potential firing, expression of A343D mutant KCNQ2 and GFP had no effect on action potential firing frequency for all current injections as it was not statistically different from the firing rates displayed by untransfected neurons or neurons expressing GFP (29.7±2.2 Hz at 100 pA injection, p>0.05, Fig. 12B–D). These data indicate that the A343D mutation completely inhibited the reduction in hippocampal neuronal excitability induced by expression of wild-type KCNQ2. Since the A343D mutation of KCNQ2 abolished axonal surface expression of heteromeric HA-KCNQ3/KCNQ2 channels (Fig. 8), these data indicate that the impaired targeting to the axonal surface by the A343D mutation of KCNQ2 has a functional consequence on how the exogenously expressed KCNQ2 subunits affect neuronal excitability.

We hypothesized that expression of KCNQ2 subunit harboring the BFNC R353G mutation would reduce the action potential firing to a similar extent as expression of wild-type subunits (Fig. 12A), since the HA-KCNQ3/KCNQ2-R353G mutant channels were enriched at the AIS and axonal surface to a similar extent as the wild-type channels (Fig. 8). To our surprise, neurons expressing R353G mutant KCNQ2 and GFP displayed a firing frequency that was indistinguishable from untransfected neurons and neurons expressing GFP (Fig. 12B–D). However, the difference in firing rates between neurons expressing R353G mutant KCNQ2 and wild-type KCNQ2 at all current injection amplitudes did not reach statistical significance (p = 0.051–0.093 at 90–200 pA current injection) except at 170 pA (p = 0.04) (Fig. 12B–D). These results indicate that the R353G mutation is sufficient to partially, but not fully, inhibit the reduction in the action potential firing rate caused by the expression of wild-type KCNQ2, even though the same mutation had no effect on axonal enrichment of surface HA-KCNQ3/KCNQ2 channels (Fig. 8). Membrane capacitance, input resistance, and resting membrane potential were unaffected by expression of GFP or KCNQ2 proteins compared to untransfected control (Table 1).

**Table 1:** Passive properties of hippocampal pyramidal neurons.

Transfection	*n*	*C*_m_ (pF)	*R*_in_ (MΩ)	*V*_m_ (mV)
Untransfected	10	42.1±2.5	937±83	−52.2±2.4
GFP	10	41.1±3.6	1137±47	−49.2±2.2
GFP + KCNQ2 WT	10	44.6±2.5	999±56	−50.7±2.0
GFP + KCNQ2 A343D	10	38.7±2.1	1049±86	−49.2±1.5
GFP + KCNQ2 R353G	9	38.0±2.5	1129±118	−50.7±0.9

*n*, number; *C*_m_, Whole-cell membrane capacitance; *R*_in_, input resistance; *V*_m_, resting membrane potential. Each value represents the Ave ± SEM.

### Coexpression of Ca^2+^-insensitive CaM modestly reduces enrichment of HA-KCNQ3/KCNQ2 channels at the axonal surface

**Figure 13:**
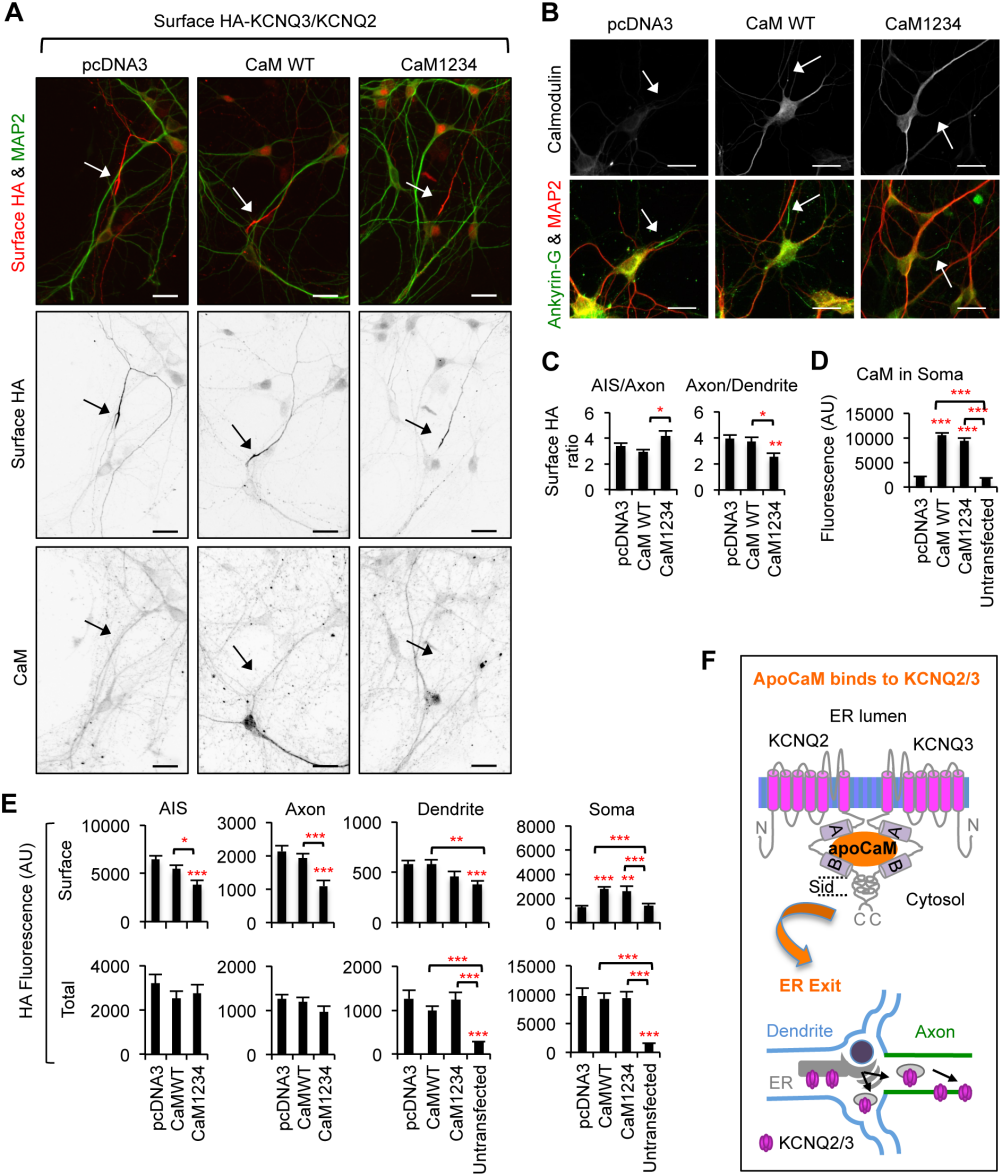
CaM1234 reduces enrichment of HA-KCNQ3/KCNQ2 channels at the axonal surface. (A) Surface expression of HA-KCNQ3/KCNQ2 channels in hippocampal neurons (DIV 7–8) cotransfected with empty vector (pcDNA3), CaM wild-type (WT) or Ca^2+^-insensitive mutant CaM (CaM1234). Endogenous and transfected CaM proteins were immunostained with anti-CaM antibodies (CaM, lower inverted images). HA-KCNQ3/KCNQ2 channels were enriched at the axonal surface in the presence of CaM WT or CaM1234. Arrows mark the main axon. Scale bars are 20 µm. (B) Overexpression of CaM WT or CaM1234 did not grossly affect neuronal polarity as indicated by immunostaining of the somatodendritic marker MAP2 and the AIS marker ankryin-G. Arrows indicate the AIS. Scale bars are 20 µm. (C) The surface “Axon/Dendrite” ratio was reduced by 33% by coexpression with CaM1234 (n = 21) compared to coexpression with CaM WT (n = 33) or empty vector pcDNA3 (n = 43). The surface “AIS/Axon” ratio of CD4-Q2C was increased by coexpression with CaM1234 compared to CaM WT but not pcDNA3. (D) Background subtracted, mean intensity of the CaM fluorescence in the soma of transfected and untransfected neurons (n = 23). (E) Background subtracted, mean intensity of surface and total (surface and intracellular) HA fluorescence in transfected and untransfected neurons. CaM1234 modestly decreased surface expression of HA-KCNQ3/KCNQ2 channels at the AIS and axon. (D, E) AU, arbitrary unit. Ave ± SEM (*p<0.05, **p<0.01, ***p<0.001). (F) Model by which apoCaM interaction with helices A and B of KCNQ2 and KCNQ3 is critical for trafficking of KCNQ2/KCNQ3 channels from the ER to the axonal surface.

Coexpression of Ca^2+^-insensitive CaM has been reported to moderately increase the ER retention of KCNQ2 by 20% in HEK293T cells [24], [26], suggesting that enrichment of KCNQ channels at the axonal surface may be regulated by Ca^2+^-bound CaM. To test this, we performed surface immunostaining of HA-KCNQ3/KCNQ2 channels in cultured hippocampal neurons that were cotransfected with either wild-type CaM or dominant-negative mutant CaM1234 (Fig. 13). Although CaM1234 is unable to bind Ca^2+^ [27], it has been shown to associate with KCNQ2 [22]. Given that CaM is abundantly expressed in cultured hippocampal neurons at 5 DIV, which is when transfections were performed (Fig. 1B), we hypothesized that coexpression of CaM1234 would displace endogenous CaM and make the CaM complex Ca^2+^-insensitive [27].

As previously demonstrated (Fig. 1D–E, 8) [18], HA-KCNQ3/KCNQ2 channels were preferentially concentrated at the AIS and distal axons compared to the soma and dendrites in neurons cotransfected with control plasmid pcDNA3 (Fig. 13A, C, E). Coexpression of CaM1234 moderately decreased their surface expression at the AIS and distal axons but not at dendrites (Fig. 13A, D–E), leading to a 33% reduction in the surface “Axon/Dendrite” ratio (CaM1234 = 2.5±0.3) compared to control plasmid (pcDNA3 = 3.9±0.3, Fig. 13C). In contrast, coexpression of wild-type CaM had no effect on the surface expression of HA-KCNQ3/KCNQ2 channels at the AIS, axon, or dendrites (Fig. 13A, C–E). Interestingly, their surface but not intracellular expression in the soma was enhanced by coexpression with wild-type CaM or CaM1234 compared to pcDNA3 control (Fig. 13D–E). Notably, coexpression of wild-type CaM and CaM1234 had no apparent effect on neuronal polarity as evidenced by strong immunolabeling of MAP2 at the soma and dendrites as well as ankyrin-G at the AIS (Fig. 13B). Since the surface “Axon/Dendrite” ratio of HA-KCNQ3/KCNQ2 channels was not reduced to 1 by coexpression of CaM1234 (Fig. 13C), our findings suggest that interaction with apoCaM but not Ca^2+^-bound CaM is necessary for preferential targeting of HA-KCNQ3/KCNQ2 channels to the axonal surface (Fig. 13F).

## Discussion

The long C-terminal tail of KCNQ2 is a multi-modal region [50], [51] that mediates assembly [52], [53], localization at the AIS and distal axon [6], [18], [20], trafficking [24], [26], and interaction with multiple signaling and adaptor proteins [22], [23], [54]–[57]. Based on our new findings, we propose that CaM binding to the IQ motif of the KCNQ2 C-terminal tail is required for preferential targeting of CD4-Q2C chimeric proteins and intact KCNQ2/KCNQ3 channels to the axonal surface in hippocampal neurons and mediates their trafficking from the ER to the axon.

### The role of CaM in the enrichment of KCNQ channels at the axonal surface

The L339R, I340E, and A343D mutations at the IQ motif of KCNQ2 have been shown to disrupt KCNQ2 interaction with CaM [24], [26]. We have shown that these same mutations abolish CaM binding to CD4-Q2C chimeric proteins as well as their axonal surface expression (Fig. 2). Although CaM can still interact with KCNQ3 subunits [23], [58], the A343D mutation, which abolished CaM binding to KCNQ2 (Fig. 8A), prevented surface and intracellular expression of intact HA-KCNQ3/KCNQ2 channels at the AIS and distal axons (Fig. 8–9), consistent with a recent study reporting altered neuronal distribution of heteromeric channels by the I340A mutation [59]. In support of our trafficking results, transient expression of wild-type KCNQ2 significantly reduced action potential firing rates whereas expression of A343D mutant KCNQ2 did not (Fig. 12). These correlative findings suggest that enrichment of CD4-Q2C and HA-KCNQ3/KCNQ2 channels at the axonal surface requires CaM interaction with the IQ motif of KCNQ2.

In contrast, the BFNC R353G mutation located distal to the IQ motif modestly reduced but did not abolish CaM binding to KCNQ2 (Fig. 8A) and had minimal effect on the polarized axonal surface expression of HA-KCNQ3/KCNQ2 channels (Fig. 8). Since the R353G mutation in KCNQ2 reduces CaM binding to heteromeric KCNQ2/KCNQ3 channels only by 20% [24], [26], our results suggest that a greater degree of impairment in CaM binding to KCNQ2 is needed to prevent targeting of heteromeric channels to the axonal surface. Recently, the S511D mutation in KCNQ2 has been shown to inhibit CaM binding without affecting surface expression of homomeric channels and KCNQ2/KCNQ3 channels in non-neuronal cells [60]. However, the S511D mutation does not restore the surface expression of I340E and A343D mutant channels [60], which is consistent with our findings that KCNQ2 mutants deficient in CaM binding not only block axonal surface expression of CD4-Q2C proteins and HA-KCNQ3/KCNQ2 channels but also reduce their somatodendritic surface expression (Fig. 2, 8). Curiously, expression of R353G mutant KCNQ2 did not reduce action potential firing frequency to the same extent as expression of wild-type KCNQ2 (Fig. 12) although HA-KCNQ3/KCNQ2-R353G mutant channels were localized to the axonal surface to a similar extent as the wild-type channels (Fig. 8). This partial effect in neuronal excitability (Fig. 12) could be due to the reduced M-current density of R353G mutant KCNQ2 channels caused by their lower affinity to Phosphatidylinositol 4,5-bisphosphate (PIP_2_) [61], an essential cofactor for M-current function [62]–[64].

We have also observed that the I340E mutation abolished the trafficking of CD4-Q2C proteins from the ER to the axons and dendrites (Fig. 7). Consistent with this result, the A343D mutation but not R353G mutation in KCNQ2 severely inhibited the trafficking of HA-KCNQ3/KCNQ2 channels from the ER to the AIS and distal axons (Fig. 11, Figure S5) where the CD4-KDEL proteins containing the ER retention signal were absent (Fig. 10A). Interestingly, wild-type and all mutant HA-KCNQ3/KCNQ2 channels were similarly distributed to the dendrites over the 8 hr time course following BFA removal (Fig. 11, Figure S5) and colocalized with the CD4-KDEL proteins (Fig. 10A). Such a distribution likely represents the diffusion and/or retention of the channels in the continuous network of the ER from the soma to the dendrites [45], [47] as well as golgi outposts [65] in hippocampal neurons. Interestingly, the A343D mutation but not the R353G mutation reduced dendritic surface expression of KCNQ2 (Fig. 8), suggesting that the A343D mutation may also hinder channel exit from the ER to the plasma membrane in dendrites. These results collectively support the idea that disruption of CaM binding to the IQ motif of KCNQ2 retains intact heteromeric channels in the ER of cultured hippocampal neurons.

Importantly, coexpression of CaM1234 modestly reduced but did not abolish enrichment of HA-KCNQ3/KCNQ2 channels at the axonal surface (Fig. 13), suggesting that KCNQ2 binding to apoCaM but not Ca^2+^-bound CaM is required for targeting the channels to the axonal surface. Since CaM regulates the activity of many key ion channels and signaling proteins that modulate neuronal excitability [66]–[71], the effect of CaM1234 could be indirect. However, our results are consistent with the previous findings in HEK293T cells that CaM1234 modestly increases ER retention of wild-type KCNQ2 by 20% [24], [26]. Given that KCNQ channel function can be potentiated in a Ca^2+^-dependent manner during activity-dependent, homeostatic intrinsic plasticity in hippocampal CA1 pyramidal neurons [72], the interaction between Ca^2+^-bound CaM and helices B of KCNQ2 and KCNQ3 [22]–[25], [39], [58], [73] may mediate exocytosis or stabilization of KCNQ channels at the axonal surface upon Ca^2+^ influx, in addition to Ca^2+^-dependent modulation of their current [39].

Considering a very low level of intracellular Ca^2+^ in dissociated hippocampal cultured neurons at rest [74], we propose that apoCaM proteins bind simultaneously to helices A and B of KCNQ2 and KCNQ3 at the ER, with the IQ motif in helix A of KCNQ2 being the dominant domain for apoCaM interaction (Fig. 13F). Such an interaction would promote association of the channels with coat protein complex II (COPII)-coated vesicles at the ER exit sites for anterograde transport to the Golgi complex [75]. After exiting the ER, KCNQ channels can be preferentially targeted to the axonal surface by multiple trafficking pathways, including direct sorting to the axons [30], [76], selective endocytosis and retention at the somatodendritic compartment [31], [77]–[79], or transcytosis from dendrites to axons [80]–[82]. The channels are then concentrated at the AIS by their interaction with ankyrin-G [6], [18], [20]. Further investigation may reveal how CaM regulates one of these trafficking pathways underlying polarized targeting to the axons.

### Comparison between CD4-Q2C proteins and HA-KCNQ3/KCNQ2 channels

We have shown that fusion of the KCNQ2 C-terminal tail was sufficient to enrich CD4 at the axonal surface (Fig. 1–2) regardless of KCNQ2 binding to ankyrin-G (Figure S2). Our finding is consistent with previous reports that polarized axonal localization of heteromeric channels does not require ankyrin-G binding of KCNQ2 and KCNQ3 [18] and occurs prior to their ankyrin-G-dependent enrichment at the AIS [6], [18], [20]. These results together suggest that CD4-Q2C chimeric proteins serve as useful trafficking reporters to dissect the mechanisms underlying polarized axonal distribution of intact KCNQ channels [18]. Indeed, we found that surface and intracellular expression of both CD4-Q2C chimeric proteins and HA-KCNQ3/KCNQ2 channels at the axons were abolished by mutations at the IQ motif of KCNQ2, which disrupt CaM binding (Fig. 2–3, 8–9).

We did however observe several distinct differences in the trafficking of CD4-Q2C proteins and HA-KCNQ3/KCNQ2 channels from the ER to the axons (Fig. 7, 11, Figure S5). In comparison to wild-type CD4-Q2C proteins, the I340E mutant CD4-Q2C proteins trafficked from the soma to the dendrites to a lesser extent during the 8 hr time course following BFA removal (Fig. 7), whereas similar amounts of wild-type and A343D mutant heteromeric channels appeared at the dendrites (Fig. 11, Figure S5). In contrast to the I340E mutant CD4-Q2C proteins, which were mostly absent from the AIS during the 8 hr time course (Fig. 7), a modest level of A343D mutant channels was detected at the AIS (Fig. 11, Figure S5). These trafficking differences could be due to channel retention in the smooth ER cisterns found at the proximal AIS [45], [46], [83], and/or due to the presence of KCNQ3, the dominant subunit that mediates KCNQ2/KCNQ3 channel localization to the AIS by binding to ankyrin-G [6], [18], [20].

In addition, the R353G mutation abolished polarized targeting of CD4-Q2C to the axonal surface by increasing dendritic surface expression (Fig. 4), whereas the same mutation had no effect on axonal enrichment of surface HA-KCNQ3/KCNQ2 channels (Fig. 8). Furthermore, the R353G mutation reduced trafficking of CD4-Q2C from the ER to the axons by half compared to the wild type (Fig. 7), whereas this mutation had no effect on the trafficking of heteromeric channels (Fig. 11, Figure S5). These results together indicate that the trafficking of CD4-Q2C protein and intact HA-KCNQ3/KCNQ2 channels to the axonal surface may not be equivalent. Despite the wide application of CD4 or CD8 chimeric proteins to understand polarized trafficking of endogenous protein complexes to axons or dendrites [18], [30], [31], [77], [84], the results from these chimeric proteins should be interpreted with caution and repeated using intact proteins.

### Physiological significance of CaM-dependent enrichment of KCNQ channels at the axonal surface

Axonal rather than somatic KCNQ channels have been shown to suppress action potential firing in hippocampal CA1 neurons [14], [17]. We have shown that expression of wild-type KCNQ2 but not A343D mutant KCNQ2 allows robust axonal surface expression of HA-KCNQ3 (Fig. 8). Accordingly, our results from whole-cell patch clamp recordings have demonstrated that expression of wild-type KCNQ2 but not A343D mutant KCNQ2 significantly dampened action potential firing rate in cultured hippocampal neurons (Fig. 12). These results reflect the differences in the axonal enrichment of wild-type and A343D mutant channels, further supporting a critical role for CaM interaction with the IQ motif of KCNQ2 in regulating axonal surface expression of intact KCNQ channels. Consistent with our finding, dissociation of CaM from KCNQ2 or decreasing CaM levels has been shown to decrease hippocampal M-current and increase neuronal excitability [23], [85]. Recently, phosphorylation of KCNQ2 by protein kinase C (PKC) has been shown to dissociate CaM from KCNQ2 channels, leading to a reduced affinity to PIP_2_ and suppression of M-current [61]. Since A-kinase-anchoring protein (AKAP) 79 [54], [86] regulates CaM binding to KCNQ2 [58] and serves as an adaptor protein for CaM and PKC [87], [88], the mechanism responsible for CaM-mediated trafficking of KCNQ channels to the axonal surface may well converge with the ability of CaM to modulate PKC-dependent inhibition of M-current via AKAP79/150 [61], [89] and PIP_2_ [61]. Considering the modest effect of CaM1234 on the axonal surface expression of HA-KCNQ3/KCNQ2 channels (Fig. 13), CaM bound to KCNQ2 may act as a Ca^2+^ sensor for modulating not only the M-current, but also the channel density at the axonal membrane.

Recent studies have identified that *de novo* mutations in helices A and B of KCNQ2 are associated with neonatal epileptic encephalopathy [90]–[92] including drug-resistant Ohtahara syndrome [93]. Since inhibition of CaM binding to the IQ motif of KCNQ2 completely blocks the targeting of heteromeric HA-KCNQ3/KCNQ2 channels from the ER to the axonal surface (Fig. 8–11), the KCNQ2 subunits with *de novo* [90]–[92] and BFNC [15] mutations in the IQ motif would likely exert a dominant negative effect by forming channels with KCNQ3 and retaining them in the ER. Complete lack of functional KCNQ channels at the axonal surface would therefore lead to burst and spontaneous firing of action potentials [10], [14] and enhanced seizure susceptibility [8], [94]. Thus, a deeper understanding of how CaM controls anterograde trafficking of KCNQ channels from the ER is expected to foster development of novel therapeutics that can enhance their axonal surface expression. Given that KCNQ channels are also implicated in hippocampal development [8], chronic inflammatory and neuropathic pain [95], anxiety [96], mania [97], and addiction [98], this novel therapeutic approach to increase KCNQ surface expression in combination with KCNQ agonist ezogabine/retigabine [16], [99] may result in an efficacious therapy for a variety of neurologic disorders.

## Supporting Information

Figure S1 **Enrichment of HA-KCNQ3/KCNQ2** **at the axon originated from a dendrite.** Surface immunostaining in hippocampal neurons (DIV 7) revealed that surface HA-KCNQ3/KCNQ2 channels (inverted image) were enriched on a MAP2-negative neurite that originates directly from a proximal dendrite. Camera lucida drawings of the neuronal images in the left show the soma and dendrites (gray) and an axon (black). Arrows mark the main axon. Scale bars are 20 µm.
(TIF)

Figure S2 **Enrichment of CD4-Q2C deficient in ankyrin-G binding at the axonal surface.** (A) Schematic drawing (not to scale) of a human KCNQ2 subunit (accession #Y15065) showing the ankyrin-G binding domain [6], CD4 alone, and CD4-Q2C. Mutations in the underlined amino acids have been shown to abolish ankyrin-G binding to KCNQ2 [6]. (B) Permeabilized immunostaining was performed in cultured hippocampal neurons (DIV 7) for the AIS marker ankryin-G and the somatodendritic marker MAP2. The dashed line marks the beginning of the axon. The bracket marks the AIS. The AIS originated from the soma (left panel) or a dendrite (right panel) started at 4.4±2.6 µm and ended at 29.8±0.7 µm from the beginning of the axon (n = 8), consistent with the previous reports on the AIS length to be about 30 µm [33]–[35]. (C) Representative inverted images of surface CD4-Q2C wild type (WT), or CD4-Q2C with E810A/D812A mutation in ankyrin-G binding motif (AIS). Camera lucida drawings (lower) of the neuronal images (upper) show the soma and dendrites (gray) and an axon (black). (D) Overlay images of the insets from Figure S2C show MAP2 (red) and surface CD4-Q2C (green). (B–D) Scale bars: 20 µm. (E) The surface “AIS/Axon” and “Axon/Dendrite” ratios were determined as previously described [18], [30] by obtaining background-subtracted mean surface CD4 fluorescence intensity of the axon between 0–30 µm (AIS) and between 50–80 µm (axon) from the beginning of the axon and the major primary dendrites. The “Axon/Dendrite” ratio shows that both WT (n = 13) and AIS mutant CD4-Q2C proteins (n = 13) were preferentially targeted to the axonal surface compared to non-polarized CD4 proteins (n = 8). The “AIS/Axon” ratio reveals that enrichment of CD4-Q2C at the AIS surface is blocked by the E810A/D812A mutation. Ave ± SEM (*p<0.05, **p<0.01, ***p<0.001).
(TIF)

Figure S3 **Identification of axons and dendrites in neurons transfected with CD4-Q2C.** Permeabilized immunostaining was performed in hippocampal neurons for the AIS using anti-phospho IκBα Ser32 (14D4) antibody to identify the axon after surface immunostaining for CD4-Q2C wild-type (WT) or mutant proteins (L339R, I340E, and A343D) was completed in Fig. 2C. Inverted images (lower) show the phospho IκBα Ser32 (14D4) immunostaining in the insets of the GFP-transfected neurons (middle). Camera lucida drawings (upper) were constructed from the inverted gray-scale images of the GFP-transfected neurons (middle) in which the axons were traced in black. Arrows indicate the AIS. Scale bars are 20 µm.
(TIF)

Figure S4 **Identification of axons and dendrites in neurons transfected with HA-KCNQ3/KCNQ2.** Permeabilized immunostaining was performed in hippocampal neurons for the AIS marker phospho IκBα Ser32 (14D4) to identify the axon after surface immunostaining for HA-KCNQ3/KCNQ2 wild-type WT or mutant (A343D and R353G) was completed in Fig. 8B. Inverted images (lower) show the AIS marker phospho IκBα Ser32 (14D4) immunostaining in the insets of the GFP-transfected neurons (middle). Camera lucida drawings (upper) were constructed from the inverted gray-scale image of the GFP-transfected neurons (middle) in which the axons were traced in black. Arrows indicate the AIS. Scale bars are 20 µm.
(TIF)

Figure S5 **Pulse-chase assay of HA-KCNQ3/KCNQ2** **channels from the ER.** Representative images of wild-type (WT) or mutant (A343D and R353G) HA-KCNQ3/KCNQ2 channels at 0, 4, and 8 hr after BFA washout in hippocampal neurons cotransfected with GFP (green). The axon was identified by immunostaining with the AIS marker phospho IκBα Ser32 (14D4). Camera lucida drawings (middle) of the GFP-transfected neurons (upper) show the soma and dendrites (gray) and an axon (black). Scale bars in the upper and middle panels are 20 µm. The small lower panels are representative inverted images of HA-KCNQ3/KCNQ2 at the AIS (AIS), distal axons (A), and dendrites (D) in transfected neurons (insets). Scale bars of the small lower panels are 10 µm.
(TIF)

## References

[1] Brown DA, Passmore GM (2009) Neural KCNQ (Kv7) channels. Br J Pharmacol 156: 1185–1195. 19298256 10.1111/j.1476-5381.2009.00111.x PMC2697739

[2] Wang HS, Pan Z, Shi W, Brown BS, Wymore RS, et al (1998) KCNQ2 and KCNQ3 potassium channel subunits: molecular correlates of the M-channel. Science 282: 1890–1893. 9836639 10.1126/science.282.5395.1890

[3] Cooper EC, Harrington E, Jan YN, Jan LY (2001) M channel KCNQ2 subunits are localized to key sites for control of neuronal network oscillations and synchronization in mouse brain. J Neurosci 21: 9529–9540. 11739564 10.1523/JNEUROSCI.21-24-09529.2001 PMC6763050

[4] Roche JP, Westenbroek R, Sorom AJ, Hille B, Mackie K, et al (2002) Antibodies and a cysteine-modifying reagent show correspondence of M current in neurons to KCNQ2 and KCNQ3 K+ channels. Br J Pharmacol 137: 1173–1186. 12466226 10.1038/sj.bjp.0704989 PMC1573614

[5] Devaux JJ, Kleopa KA, Cooper EC, Scherer SS (2004) KCNQ2 is a nodal K+ channel. J Neurosci 24: 1236–1244. 14762142 10.1523/JNEUROSCI.4512-03.2004 PMC6793582

[6] Pan Z, Kao T, Horvath Z, Lemos J, Sul JY, et al (2006) A common ankyrin-G-based mechanism retains KCNQ and NaV channels at electrically active domains of the axon. J Neurosci 26: 2599–2613. 16525039 10.1523/JNEUROSCI.4314-05.2006 PMC6675151

[7] Brown DA, Constanti A, Adams PR (1981) Slow cholinergic and peptidergic transmission in sympathetic ganglia. Fed Proc 40: 2625–2630. 6268462

[8] Peters HC, Hu H, Pongs O, Storm JF, Isbrandt D (2005) Conditional transgenic suppression of M channels in mouse brain reveals functions in neuronal excitability, resonance and behavior. Nat Neurosci 8: 51–60. 15608631 10.1038/nn1375

[9] Gu N, Vervaeke K, Hu H, Storm JF (2005) Kv7/KCNQ/M and HCN/h, but not KCa2/SK channels, contribute to the somatic medium after-hyperpolarization and excitability control in CA1 hippocampal pyramidal cells. J Physiol 566: 689–715. 15890705 10.1113/jphysiol.2005.086835 PMC1464792

[10] Yue C, Yaari Y (2004) KCNQ/M channels control spike afterdepolarization and burst generation in hippocampal neurons. J Neurosci 24: 4614–4624. 15140933 10.1523/JNEUROSCI.0765-04.2004 PMC6729392

[11] Yue C, Yaari Y (2006) Axo-Somatic and Apical Dendritic Kv7/M Channels Differentially Regulate the Intrinsic Excitability of Adult Rat CA1 Pyramidal Cells. J Neurophysiol. 10.1152/jn.01333.2005 16495357

[12] Tzingounis AV, Nicoll RA (2008) Contribution of KCNQ2 and KCNQ3 to the medium and slow afterhyperpolarization currents. Proc Natl Acad Sci U S A 105: 19974–19979. 19060215 10.1073/pnas.0810535105 PMC2604953

[13] Tzingounis AV, Heidenreich M, Kharkovets T, Spitzmaul G, Jensen HS, et al (2010) The KCNQ5 potassium channel mediates a component of the afterhyperpolarization current in mouse hippocampus. Proc Natl Acad Sci U S A 107: 10232–10237. 20534576 10.1073/pnas.1004644107 PMC2890451

[14] Shah MM, Migliore M, Valencia I, Cooper EC, Brown DA (2008) Functional significance of axonal Kv7 channels in hippocampal pyramidal neurons. Proc Natl Acad Sci U S A 105: 7869–7874. 18515424 10.1073/pnas.0802805105 PMC2408483

[15] Maljevic S, Wuttke TV, Seebohm G, Lerche H (2010) KV7 channelopathies. Pflugers Arch 460: 277–288. 20401729 10.1007/s00424-010-0831-3

[16] Gunthorpe MJ, Large CH, Sankar R (2012) The mechanism of action of retigabine (ezogabine), a first-in-class K+ channel opener for the treatment of epilepsy. Epilepsia 53: 412–424. 22220513 10.1111/j.1528-1167.2011.03365.x

[17] Shah MM, Migliore M, Brown DA (2011) Differential effects of Kv7 (M-) channels on synaptic integration in distinct subcellular compartments of rat hippocampal pyramidal neurons. J Physiol 589: 6029–6038. 22041186 10.1113/jphysiol.2011.220913 PMC3245855

[18] Chung HJ, Jan YN, Jan LY (2006) Polarized axonal surface expression of neuronal KCNQ channels is mediated by multiple signals in the KCNQ2 and KCNQ3 C-terminal domains. Proc Natl Acad Sci U S A 103: 8870–8875. 16735477 10.1073/pnas.0603376103 PMC1472242

[19] Clark BD, Goldberg EM, Rudy B (2009) Electrogenic tuning of the axon initial segment. Neuroscientist 15: 651–668. 20007821 10.1177/1073858409341973 PMC2951114

[20] Rasmussen HB, Frokjaer-Jensen C, Jensen CS, Jensen HS, Jorgensen NK, et al (2007) Requirement of subunit co-assembly and ankyrin-G for M-channel localization at the axon initial segment. J Cell Sci 120: 953–963. 17311847 10.1242/jcs.03396

[21] Song AH, Wang D, Chen G, Li Y, Luo J, et al (2009) A selective filter for cytoplasmic transport at the axon initial segment. Cell 136: 1148–1160. 19268344 10.1016/j.cell.2009.01.016

[22] Wen H, Levitan IB (2002) Calmodulin is an auxiliary subunit of KCNQ2/3 potassium channels. J Neurosci 22: 7991–8001. 12223552 10.1523/JNEUROSCI.22-18-07991.2002 PMC6758071

[23] Yus-Najera E, Santana-Castro I, Villarroel A (2002) The identification and characterization of a noncontinuous calmodulin-binding site in noninactivating voltage-dependent KCNQ potassium channels. J Biol Chem 277: 28545–28553. 12032157 10.1074/jbc.M204130200

[24] Alaimo A, Gomez-Posada JC, Aivar P, Etxeberria A, Rodriguez-Alfaro JA, et al (2009) Calmodulin activation limits the rate of KCNQ2 K+ channel exit from the endoplasmic reticulum. J Biol Chem 284: 20668–20675. 19494108 10.1074/jbc.M109.019539 PMC2742831

[25] Xu Q, Chang A, Tolia A, Minor DL Jr (2013) Structure of a Ca(2+)/CaM:Kv7.4 (KCNQ4) B-helix complex provides insight into M current modulation. J Mol Biol 425: 378–394. 23178170 10.1016/j.jmb.2012.11.023 PMC3540129

[26] Etxeberria A, Aivar P, Rodriguez-Alfaro JA, Alaimo A, Villace P, et al (2008) Calmodulin regulates the trafficking of KCNQ2 potassium channels. FASEB J 22: 1135–1143. 17993630 10.1096/fj.07-9712com

[27] Xia XM, Fakler B, Rivard A, Wayman G, Johnson-Pais T, et al (1998) Mechanism of calcium gating in small-conductance calcium-activated potassium channels. Nature 395: 503–507. 9774106 10.1038/26758

[28] Schwake M, Pusch M, Kharkovets T, Jentsch TJ (2000) Surface expression and single channel properties of KCNQ2/KCNQ3, M-type K+ channels involved in epilepsy. J Biol Chem 275: 13343–13348. 10788442 10.1074/jbc.275.18.13343

[29] Zerangue N, Malan MJ, Fried SR, Dazin PF, Jan YN, et al (2001) Analysis of endoplasmic reticulum trafficking signals by combinatorial screening in mammalian cells. Proc Natl Acad Sci U S A 98: 2431–2436. 11226256 10.1073/pnas.051630198 PMC30155

[30] Gu C, Jan YN, Jan LY (2003) A conserved domain in axonal targeting of Kv1 (Shaker) voltage-gated potassium channels. Science 301: 646–649. 12893943 10.1126/science.1086998

[31] Fache MP, Moussif A, Fernandes F, Giraud P, Garrido JJ, et al (2004) Endocytotic elimination and domain-selective tethering constitute a potential mechanism of protein segregation at the axonal initial segment. J Cell Biol 166: 571–578. 15302857 10.1083/jcb.200312155 PMC2172218

[32] Sanchez-Ponce D, DeFelipe J, Garrido JJ, Munoz A (2012) Developmental expression of Kv potassium channels at the axon initial segment of cultured hippocampal neurons. PLoS One 7: e48557. 23119056 10.1371/journal.pone.0048557 PMC3485302

[33] Kuba H, Oichi Y, Ohmori H (2010) Presynaptic activity regulates Na(+) channel distribution at the axon initial segment. Nature 465: 1075–1078. 20543825 10.1038/nature09087

[34] Duflocq A, Chareyre F, Giovannini M, Couraud F, Davenne M (2011) Characterization of the axon initial segment (AIS) of motor neurons and identification of a para-AIS and a juxtapara-AIS, organized by protein 4.1B. BMC Biol 9: 66. 21958379 10.1186/1741-7007-9-66 PMC3198992

[35] Grubb MS, Burrone J (2010) Activity-dependent relocation of the axon initial segment fine-tunes neuronal excitability. Nature 465: 1070–1074. 20543823 10.1038/nature09160 PMC3196626

[36] Meijering E, Jacob M, Sarria JC, Steiner P, Hirling H, et al (2004) Design and validation of a tool for neurite tracing and analysis in fluorescence microscopy images. Cytometry A 58: 167–176. 15057970 10.1002/cyto.a.20022

[37] Lewis TL Jr, Mao T, Svoboda K, Arnold DB (2009) Myosin-dependent targeting of transmembrane proteins to neuronal dendrites. Nat Neurosci 12: 568–576. 19377470 10.1038/nn.2318 PMC2937175

[38] Chung HJ, Qian X, Ehlers M, Jan YN, Jan LY (2009) Neuronal activity regulates phosphorylation-dependent surface delivery of G protein-activated inwardly rectifying potassium channels. Proc Natl Acad Sci U S A 106: 629–634. 19118198 10.1073/pnas.0811615106 PMC2613039

[39] Gamper N, Shapiro MS (2003) Calmodulin mediates Ca2+-dependent modulation of M-type K+ channels. J Gen Physiol 122: 17–31. 12810850 10.1085/jgp.200208783 PMC2234471

[40] Desai NS, Rutherford LC, Turrigiano GG (1999) Plasticity in the intrinsic excitability of cortical pyramidal neurons. Nat Neurosci 2: 515–520. 10448215 10.1038/9165

[41] Weber YG, Geiger J, Kampchen K, Landwehrmeyer B, Sommer C, et al (2006) Immunohistochemical analysis of KCNQ2 potassium channels in adult and developing mouse brain. Brain Res. 10.1016/j.brainres.2006.01.023 16500630

[42] Geiger J, Weber YG, Landwehrmeyer B, Sommer C, Lerche H (2006) Immunohistochemical analysis of KCNQ3 potassium channels in mouse brain. Neurosci Lett. 10.1016/j.neulet.2006.02.017 16513263

[43] Richards MC, Heron SE, Spendlove HE, Scheffer IE, Grinton B, et al (2004) Novel mutations in the KCNQ2 gene link epilepsy to a dysfunction of the KCNQ2-calmodulin interaction. J Med Genet 41: e35. 14985406 10.1136/jmg.2003.013938 PMC1735682

[44] Fujiwara T, Oda K, Yokota S, Takatsuki A, Ikehara Y (1988) Brefeldin A causes disassembly of the Golgi complex and accumulation of secretory proteins in the endoplasmic reticulum. J Biol Chem 263: 18545–18552. 3192548

[45] Krijnse-Locker J, Parton RG, Fuller SD, Griffiths G, Dotti CG (1995) The organization of the endoplasmic reticulum and the intermediate compartment in cultured rat hippocampal neurons. Mol Biol Cell 6: 1315–1332. 8573789 10.1091/mbc.6.10.1315 PMC301290

[46] Terasaki M, Slater NT, Fein A, Schmidek A, Reese TS (1994) Continuous network of endoplasmic reticulum in cerebellar Purkinje neurons. Proc Natl Acad Sci U S A 91: 7510–7514. 7519781 10.1073/pnas.91.16.7510 PMC44431

[47] Spacek J, Harris KM (1997) Three-dimensional organization of smooth endoplasmic reticulum in hippocampal CA1 dendrites and dendritic spines of the immature and mature rat. J Neurosci 17: 190–203. 8987748 10.1523/JNEUROSCI.17-01-00190.1997 PMC6793680

[48] Terasaki M, Jaffe LA, Hunnicutt GR, Hammer JA 3rd (1996) Structural change of the endoplasmic reticulum during fertilization: evidence for loss of membrane continuity using the green fluorescent protein. Dev Biol 179: 320–328. 8903348 10.1006/dbio.1996.0263

[49] Roderick HL, Campbell AK, Llewellyn DH (1997) Nuclear localisation of calreticulin in vivo is enhanced by its interaction with glucocorticoid receptors. FEBS Lett 405: 181–185. 9089287 10.1016/s0014-5793(97)00183-x

[50] Haitin Y, Attali B (2008) The C-terminus of Kv7 channels: a multifunctional module. J Physiol 586: 1803–1810. 18218681 10.1113/jphysiol.2007.149187 PMC2375714

[51] Hernandez CC, Zaika O, Tolstykh GP, Shapiro MS (2008) Regulation of neural KCNQ channels: signalling pathways, structural motifs and functional implications. J Physiol 586: 1811–1821. 18238808 10.1113/jphysiol.2007.148304 PMC2375728

[52] Schwake M, Jentsch TJ, Friedrich T (2003) A carboxy-terminal domain determines the subunit specificity of KCNQ K+ channel assembly. EMBO Rep 4: 76–81. 12524525 10.1038/sj.embor.embor715 PMC1315815

[53] Maljevic S, Lerche C, Seebohm G, Alekov AK, Busch AE, et al (2003) C-terminal interaction of KCNQ2 and KCNQ3 K+ channels. J Physiol 548: 353–360. 12640002 10.1113/jphysiol.2003.040980 PMC2342851

[54] Hoshi N, Zhang JS, Omaki M, Takeuchi T, Yokoyama S, et al (2003) AKAP150 signaling complex promotes suppression of the M-current by muscarinic agonists. Nat Neurosci 6: 564–571. 12754513 10.1038/nn1062 PMC3941299

[55] Ekberg J, Schuetz F, Boase NA, Conroy SJ, Manning J, et al (2007) Regulation of the voltage-gated K(+) channels KCNQ2/3 and KCNQ3/5 by ubiquitination. Novel role for Nedd4-2. J Biol Chem 282: 12135–12142. 17322297 10.1074/jbc.M609385200

[56] Etzioni A, Siloni S, Chikvashvilli D, Strulovich R, Sachyani D, et al (2011) Regulation of neuronal M-channel gating in an isoform-specific manner: functional interplay between calmodulin and syntaxin 1A. J Neurosci 31: 14158–14171. 21976501 10.1523/JNEUROSCI.2666-11.2011 PMC6623657

[57] Regev N, Degani-Katzav N, Korngreen A, Etzioni A, Siloni S, et al (2009) Selective interaction of syntaxin 1A with KCNQ2: possible implications for specific modulation of presynaptic activity. PLoS One 4: e6586. 19675672 10.1371/journal.pone.0006586 PMC2721677

[58] Bal M, Zhang J, Hernandez CC, Zaika O, Shapiro MS (2010) Ca2+/calmodulin disrupts AKAP79/150 interactions with KCNQ (M-Type) K+ channels. J Neurosci 30: 2311–2323. 20147557 10.1523/JNEUROSCI.5175-09.2010 PMC2832802

[59] Liu W, Devaux JJ (2013) Calmodulin orchestrates the heteromeric assembly and the trafficking of KCNQ2/3 (Kv7.2/3) channels in neurons. Mol Cell Neurosci 58C: 40–52. 10.1016/j.mcn.2013.12.005 24333508

[60] Gomez-Posada JC, Aivar P, Alberdi A, Alaimo A, Etxeberria A, et al (2011) Kv7 channels can function without constitutive calmodulin tethering. PLoS One 6: e25508. 21980481 10.1371/journal.pone.0025508 PMC3182250

[61] Kosenko A, Kang S, Smith IM, Greene DL, Langeberg LK, et al (2012) Coordinated signal integration at the M-type potassium channel upon muscarinic stimulation. EMBO J 31: 3147–3156. 22643219 10.1038/emboj.2012.156 PMC3400014

[62] Suh BC, Hille B (2002) Recovery from muscarinic modulation of M current channels requires phosphatidylinositol 4,5-bisphosphate synthesis. Neuron 35: 507–520. 12165472 10.1016/s0896-6273(02)00790-0

[63] Zhang H, Craciun LC, Mirshahi T, Rohacs T, Lopes CM, et al (2003) PIP(2) activates KCNQ channels, and its hydrolysis underlies receptor-mediated inhibition of M currents. Neuron 37: 963–975. 12670425 10.1016/s0896-6273(03)00125-9

[64] Winks JS, Hughes S, Filippov AK, Tatulian L, Abogadie FC, et al (2005) Relationship between membrane phosphatidylinositol-4,5-bisphosphate and receptor-mediated inhibition of native neuronal M channels. J Neurosci 25: 3400–3413. 15800195 10.1523/JNEUROSCI.3231-04.2005 PMC6724893

[65] Horton AC, Ehlers MD (2003) Neuronal polarity and trafficking. Neuron 40: 277–295. 14556709 10.1016/s0896-6273(03)00629-9

[66] Liu XB, Murray KD (2012) Neuronal excitability and calcium/calmodulin-dependent protein kinase type II: location, location, location. Epilepsia 53 Suppl 1: 45–52. 10.1111/j.1528-1167.2012.03474.x 22612808

[67] Goraya TA, Cooper DM (2005) Ca2+-calmodulin-dependent phosphodiesterase (PDE1): current perspectives. Cell Signal 17: 789–797. 15763421 10.1016/j.cellsig.2004.12.017

[68] Zhu MX (2005) Multiple roles of calmodulin and other Ca(2+)-binding proteins in the functional regulation of TRP channels. Pflugers Arch 451: 105–115. 15924238 10.1007/s00424-005-1427-1

[69] Maylie J, Bond CT, Herson PS, Lee WS, Adelman JP (2004) Small conductance Ca2+-activated K+ channels and calmodulin. J Physiol 554: 255–261. 14500775 10.1113/jphysiol.2003.049072 PMC1664776

[70] Halling DB, Aracena-Parks P, Hamilton SL (2006) Regulation of voltage-gated Ca2+ channels by calmodulin. Sci STKE 2006: er1. 16685765 10.1126/stke.3182006er1

[71] Wayman GA, Lee YS, Tokumitsu H, Silva AJ, Soderling TR (2008) Calmodulin-kinases: modulators of neuronal development and plasticity. Neuron 59: 914–931. 18817731 10.1016/j.neuron.2008.08.021 PMC2664743

[72] Wu WW, Chan CS, Surmeier DJ, Disterhoft JF (2008) Coupling of L-type Ca2+ channels to KV7/KCNQ channels creates a novel, activity-dependent, homeostatic intrinsic plasticity. J Neurophysiol 100: 1897–1908. 18715900 10.1152/jn.90346.2008 PMC2576227

[73] Mruk K, Shandilya SM, Blaustein RO, Schiffer CA, Kobertz WR (2012) Structural insights into neuronal K+ channel-calmodulin complexes. Proc Natl Acad Sci U S A 109: 13579–13583. 22869708 10.1073/pnas.1207606109 PMC3427091

[74] Nunez JL, McCarthy MM (2009) Resting intracellular calcium concentration, depolarizing Gamma-Aminobutyric Acid and possible role of local estradiol synthesis in the developing male and female hippocampus. Neuroscience 158: 623–634. 19007865 10.1016/j.neuroscience.2008.09.061 PMC2660432

[75] Zanetti G, Pahuja KB, Studer S, Shim S, Schekman R (2012) COPII and the regulation of protein sorting in mammals. Nat Cell Biol 14: 20–28. 10.1038/ncb2390 22193160

[76] Rivera JF, Ahmad S, Quick MW, Liman ER, Arnold DB (2003) An evolutionarily conserved dileucine motif in Shal K+ channels mediates dendritic targeting. Nat Neurosci 6: 243–250. 12592409 10.1038/nn1020

[77] Garrido JJ, Fernandes F, Giraud P, Mouret I, Pasqualini E, et al (2001) Identification of an axonal determinant in the C-terminus of the sodium channel Na(v)1.2. Embo J 20: 5950–5961. 11689435 10.1093/emboj/20.21.5950 PMC125703

[78] Garrido JJ, Fernandes F, Moussif A, Fache MP, Giraud P, et al (2003) Dynamic compartmentalization of the voltage-gated sodium channels in axons. Biol Cell 95: 437–445. 14597261 10.1016/s0248-4900(03)00091-1

[79] Sampo B, Kaech S, Kunz S, Banker G (2003) Two distinct mechanisms target membrane proteins to the axonal surface. Neuron 37: 611–624. 12597859 10.1016/s0896-6273(03)00058-8

[80] Wisco D, Anderson ED, Chang MC, Norden C, Boiko T, et al (2003) Uncovering multiple axonal targeting pathways in hippocampal neurons. J Cell Biol 162: 1317–1328. 14517209 10.1083/jcb.200307069 PMC2173963

[81] Yap CC, Nokes RL, Wisco D, Anderson E, Folsch H, et al (2008) Pathway selection to the axon depends on multiple targeting signals in NgCAM. J Cell Sci 121: 1514–1525. 18411247 10.1242/jcs.022442

[82] Yap CC, Wisco D, Kujala P, Lasiecka ZM, Cannon JT, et al (2008) The somatodendritic endosomal regulator NEEP21 facilitates axonal targeting of L1/NgCAM. J Cell Biol 180: 827–842. 18299352 10.1083/jcb.200707143 PMC2265569

[83] Bas Orth C, Schultz C, Muller CM, Frotscher M, Deller T (2007) Loss of the cisternal organelle in the axon initial segment of cortical neurons in synaptopodin-deficient mice. J Comp Neurol 504: 441–449. 17701995 10.1002/cne.21445

[84] Lewis TL Jr, Mao T, Arnold DB (2011) A role for myosin VI in the localization of axonal proteins. PLoS Biol 9: e1001021. 21390300 10.1371/journal.pbio.1001021 PMC3046960

[85] Shahidullah M, Santarelli LC, Wen H, Levitan IB (2005) Expression of a calmodulin-binding KCNQ2 potassium channel fragment modulates neuronal M-current and membrane excitability. Proc Natl Acad Sci U S A 102: 16454–16459. 16263935 10.1073/pnas.0503966102 PMC1283421

[86] Cooper EC, Aldape KD, Abosch A, Barbaro NM, Berger MS, et al (2000) Colocalization and coassembly of two human brain M-type potassium channel subunits that are mutated in epilepsy. Proc Natl Acad Sci U S A 97: 4914–4919. 10781098 10.1073/pnas.090092797 PMC18332

[87] Wong W, Scott JD (2004) AKAP signalling complexes: focal points in space and time. Nat Rev Mol Cell Biol 5: 959–970. 15573134 10.1038/nrm1527

[88] Klauck TM, Faux MC, Labudda K, Langeberg LK, Jaken S, et al (1996) Coordination of three signaling enzymes by AKAP79, a mammalian scaffold protein. Science 271: 1589–1592. 8599116 10.1126/science.271.5255.1589

[89] Higashida H, Hoshi N, Zhang JS, Yokoyama S, Hashii M, et al (2005) Protein kinase C bound with A-kinase anchoring protein is involved in muscarinic receptor-activated modulation of M-type KCNQ potassium channels. Neurosci Res 51: 231–234. 15710486 10.1016/j.neures.2004.11.009

[90] Weckhuysen S, Mandelstam S, Suls A, Audenaert D, Deconinck T, et al (2012) KCNQ2 encephalopathy: emerging phenotype of a neonatal epileptic encephalopathy. Ann Neurol 71: 15–25. 22275249 10.1002/ana.22644

[91] Borgatti R, Zucca C, Cavallini A, Ferrario M, Panzeri C, et al (2004) A novel mutation in KCNQ2 associated with BFNC, drug resistant epilepsy, and mental retardation. Neurology 63: 57–65. 15249611 10.1212/01.wnl.0000132979.08394.6d

[92] Schmitt B, Wohlrab G, Sander T, Steinlein OK, Hajnal BL (2005) Neonatal seizures with tonic clonic sequences and poor developmental outcome. Epilepsy Res 65: 161–168. 16039833 10.1016/j.eplepsyres.2005.05.009

[93] Saitsu H, Kato M, Koide A, Goto T, Fujita T, et al (2012) Whole exome sequencing identifies KCNQ2 mutations in Ohtahara syndrome. Ann Neurol 72: 298–300. 22926866 10.1002/ana.23620

[94] Watanabe H, Nagata E, Kosakai A, Nakamura M, Yokoyama M, et al (2000) Disruption of the epilepsy KCNQ2 gene results in neural hyperexcitability. J Neurochem 75: 28–33. 10854243 10.1046/j.1471-4159.2000.0750028.x

[95] Munro G, Dalby-Brown W (2007) Kv7 (KCNQ) channel modulators and neuropathic pain. J Med Chem 50: 2576–2582. 17489574 10.1021/jm060989l

[96] Korsgaard MP, Hartz BP, Brown WD, Ahring PK, Strobaek D, et al (2005) Anxiolytic effects of Maxipost (BMS-204352) and retigabine via activation of neuronal Kv7 channels. J Pharmacol Exp Ther 314: 282–292. 15814569 10.1124/jpet.105.083923

[97] Dencker D, Dias R, Pedersen ML, Husum H (2008) Effect of the new antiepileptic drug retigabine in a rodent model of mania. Epilepsy Behav 12: 49–53. 18086455 10.1016/j.yebeh.2007.09.023

[98] Hansen HH, Andreasen JT, Weikop P, Mirza N, Scheel-Kruger J, et al (2007) The neuronal KCNQ channel opener retigabine inhibits locomotor activity and reduces forebrain excitatory responses to the psychostimulants cocaine, methylphenidate and phencyclidine. Eur J Pharmacol 570: 77–88. 17628530 10.1016/j.ejphar.2007.05.029

[99] Large CH, Sokal DM, Nehlig A, Gunthorpe MJ, Sankar R, et al (2012) The spectrum of anticonvulsant efficacy of retigabine (ezogabine) in animal models: implications for clinical use. Epilepsia 53: 425–436. 22221318 10.1111/j.1528-1167.2011.03364.x

