# Supplementary figures and images for: Polarized Axonal Surface Expression of Neuronal KCNQ Potassium Channels Is Regulated by Calmodulin Interaction with KCNQ2 Subunit
Source: PLoS One. 2014 Jul 31;9(7):e103655. doi: 10.1371/journal.pone.0103655 (PMC4117524; doi:10.1371/journal.pone.0103655)

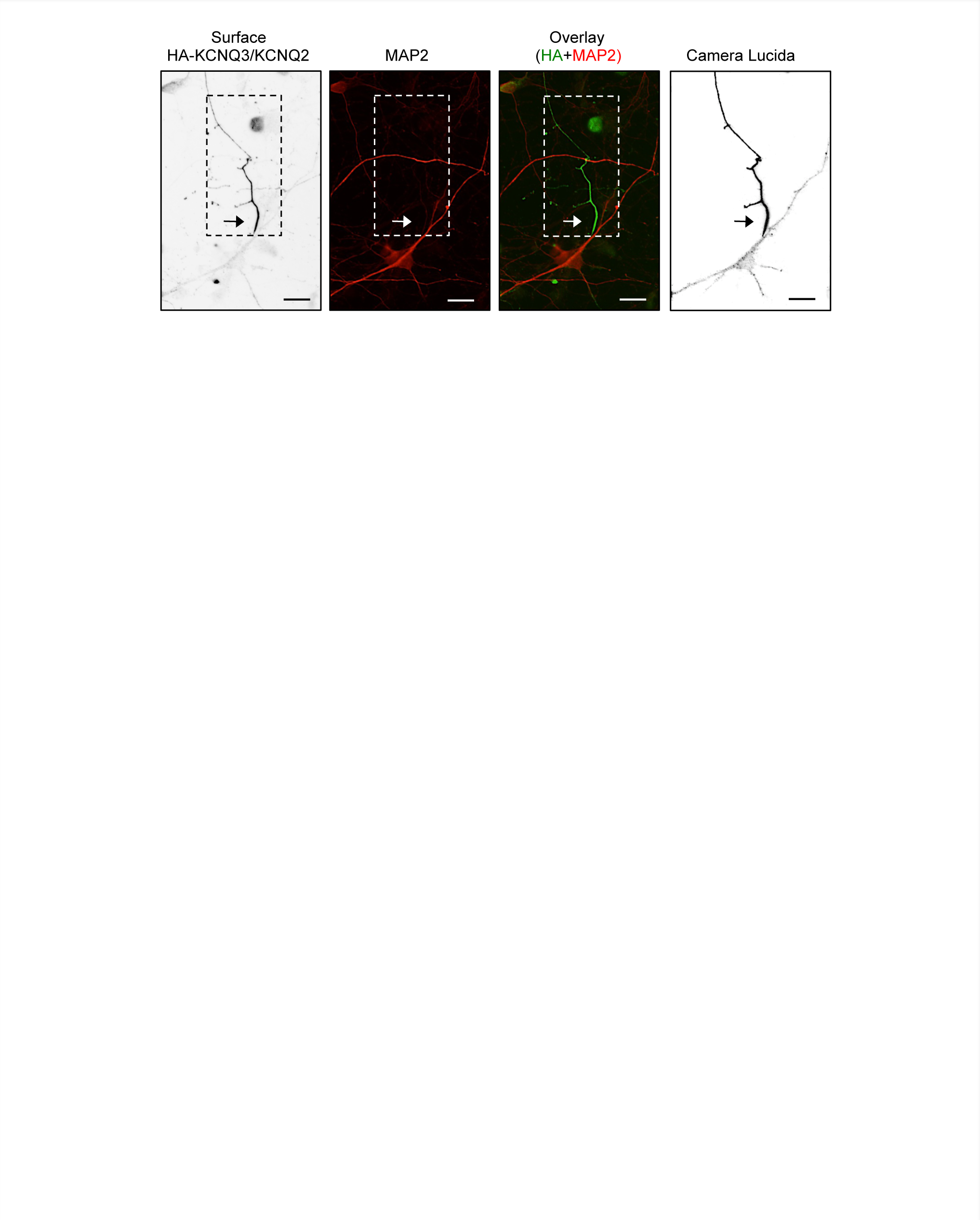

Supplement: Figure S1 — Enrichment of HA-KCNQ3/KCNQ2 at the axon originated from a dendrite. Surface immunostaining in hippocampal neurons (DIV 7) revealed that surface HA-KCNQ3/KCNQ2 channels (inverted image) were enriched on a MAP2-negative neurite that originates directly from a proximal dendrite. Camera lucida drawings of the neuronal images in the left show the soma and dendrites (gray) and an axon (black). Arrows mark the main axon. Scale bars are 20 µm. (TIF) [file pone.0103655.s001.tif]

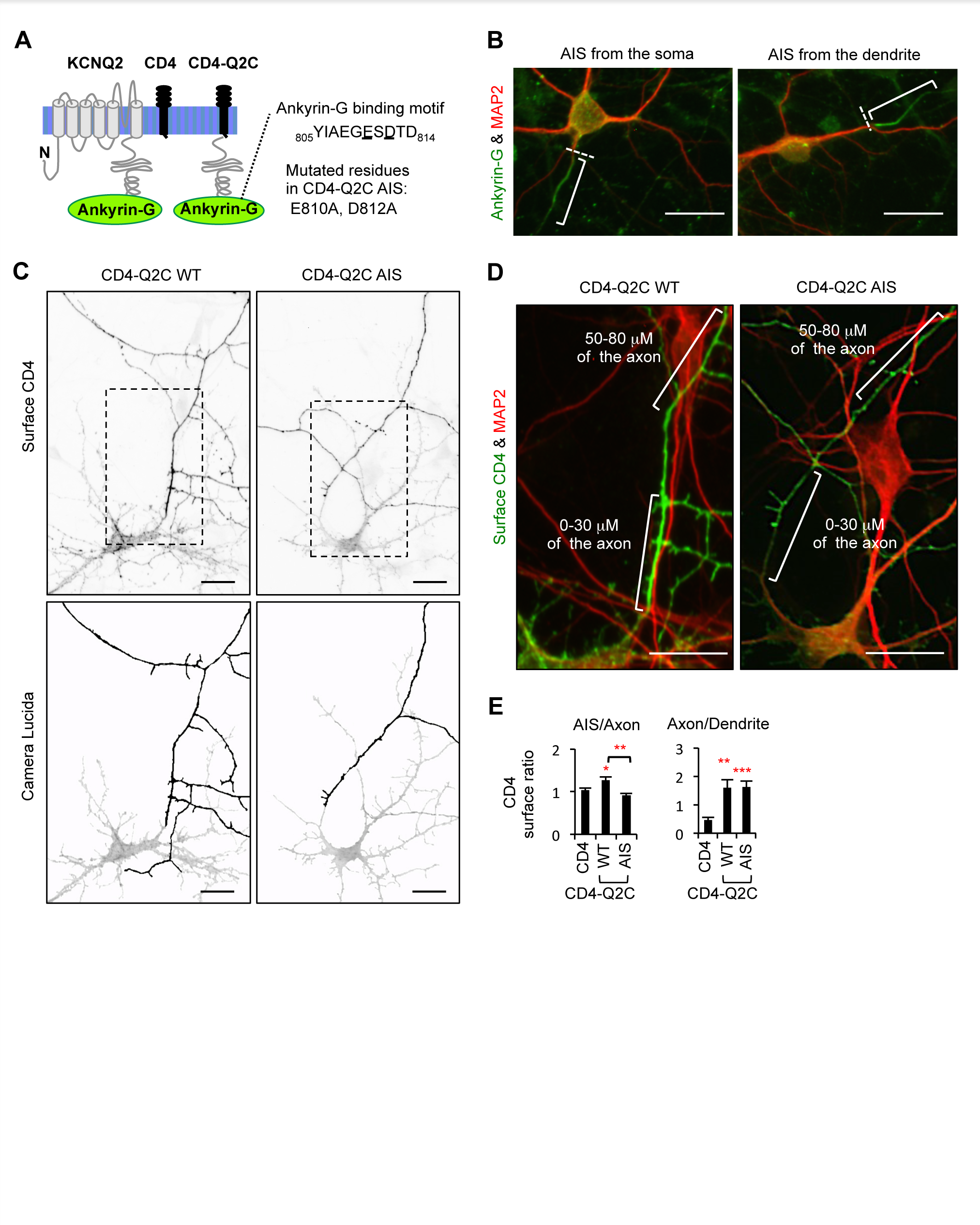

Supplement: Figure S2 — Enrichment of CD4-Q2C deficient in ankyrin-G binding at the axonal surface. (A) Schematic drawing (not to scale) of a human KCNQ2 subunit (accession #Y15065) showing the ankyrin-G binding domain [6], CD4 alone, and CD4-Q2C. Mutations in the underlined amino acids have been shown to abolish ankyrin-G binding to KCNQ2 [6]. (B) Permeabilized immunostaining was performed in cultured hippocampal neurons (DIV 7) for the AIS marker ankryin-G and the somatodendritic marker MAP2. The dashed line marks the beginning of the axon. The bracket marks the AIS. The AIS originated from the soma (left panel) or a dendrite (right panel) started at 4.4±2.6 µm and ended at 29.8±0.7 µm from the beginning of the axon (n = 8), consistent with the previous reports on the AIS length to be about 30 µm [33]–[35]. (C) Representative inverted images of surface CD4-Q2C wild type (WT), or CD4-Q2C with E810A/D812A mutation in ankyrin-G binding motif (AIS). Camera lucida drawings (lower) of the neuronal images (upper) show the soma and dendrites (gray) and an axon (black). (D) Overlay images of the insets from Figure S2C show MAP2 (red) and surface CD4-Q2C (green). (B–D) Scale bars: 20 µm. (E) The surface “AIS/Axon” and “Axon/Dendrite” ratios were determined as previously described [18], [30] by obtaining background-subtracted mean surface CD4 fluorescence intensity of the axon between 0–30 µm (AIS) and between 50–80 µm (axon) from the beginning of the axon and the major primary dendrites. The “Axon/Dendrite” ratio shows that both WT (n = 13) and AIS mutant CD4-Q2C proteins (n = 13) were preferentially targeted to the axonal surface compared to non-polarized CD4 proteins (n = 8). The “AIS/Axon” ratio reveals that enrichment of CD4-Q2C at the AIS surface is blocked by the E810A/D812A mutation. Ave ± SEM (*p<0.05, **p<0.01, ***p<0.001). (TIF) [file pone.0103655.s002.tif]

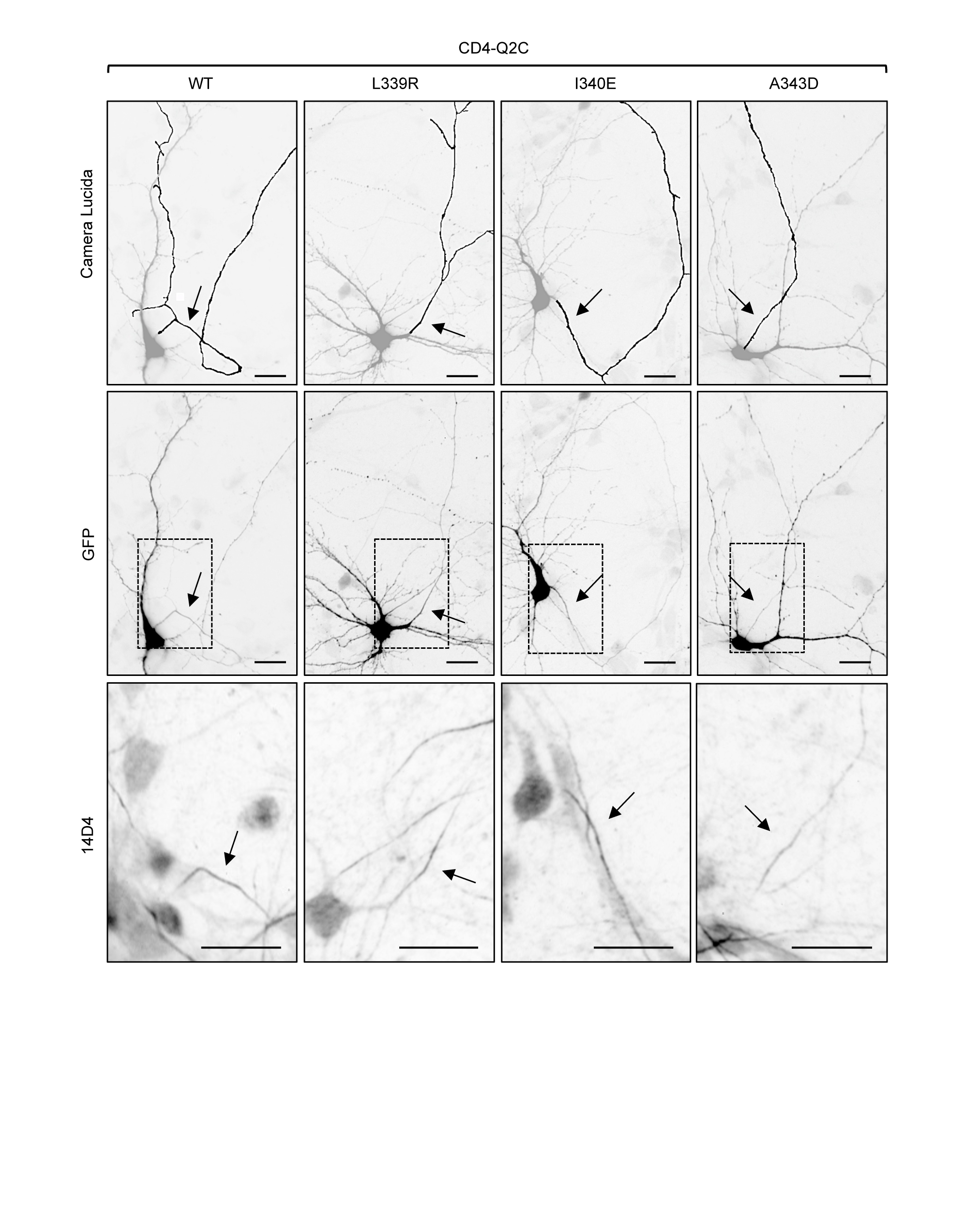

Supplement: Figure S3 — Identification of axons and dendrites in neurons transfected with CD4-Q2C. Permeabilized immunostaining was performed in hippocampal neurons for the AIS using anti-phospho IκBα Ser32 (14D4) antibody to identify the axon after surface immunostaining for CD4-Q2C wild-type (WT) or mutant proteins (L339R, I340E, and A343D) was completed in Fig. 2C. Inverted images (lower) show the phospho IκBα Ser32 (14D4) immunostaining in the insets of the GFP-transfected neurons (middle). Camera lucida drawings (upper) were constructed from the inverted gray-scale images of the GFP-transfected neurons (middle) in which the axons were traced in black. Arrows indicate the AIS. Scale bars are 20 µm. (TIF) [file pone.0103655.s003.tif]

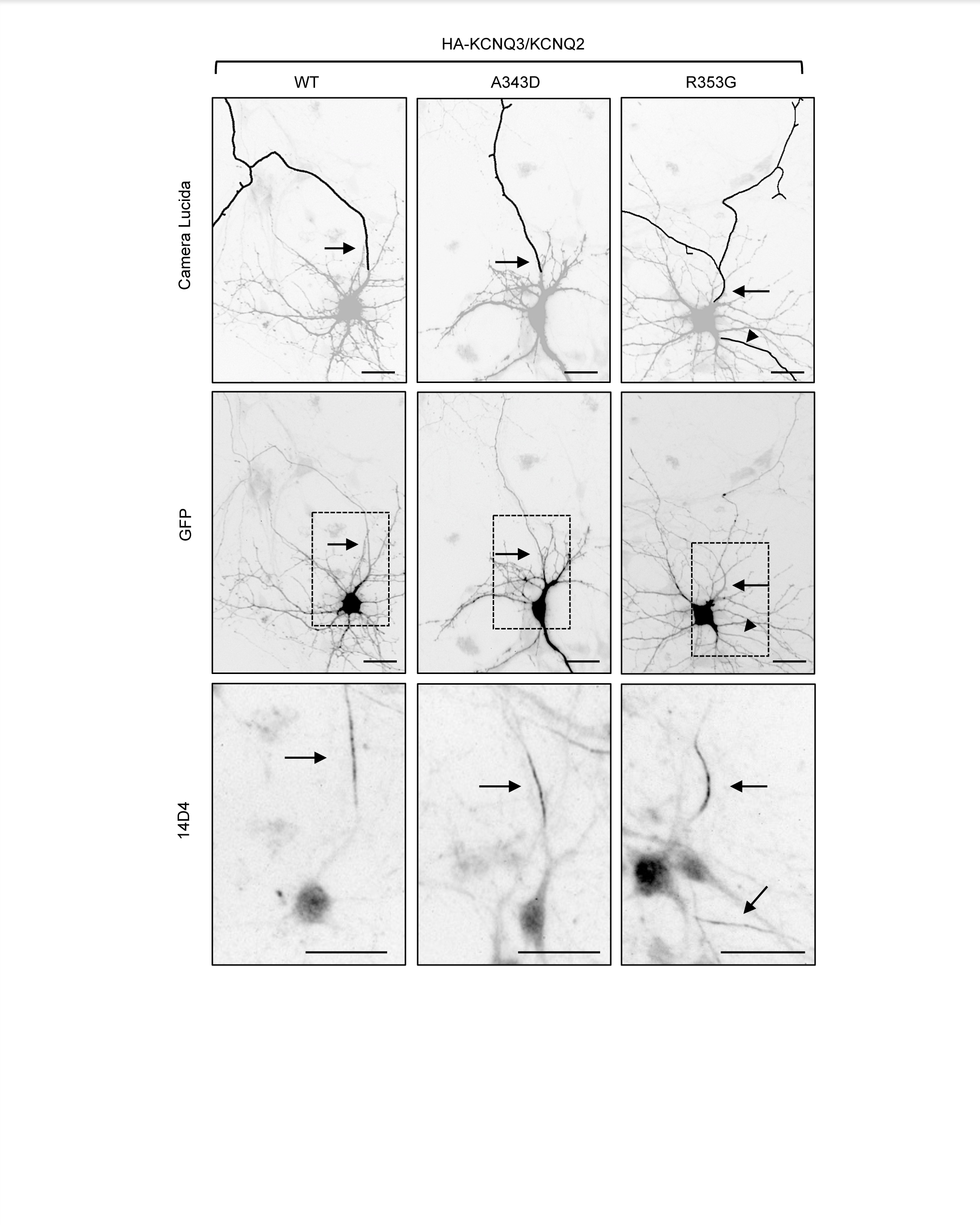

Supplement: Figure S4 — Identification of axons and dendrites in neurons transfected with HA-KCNQ3/KCNQ2. Permeabilized immunostaining was performed in hippocampal neurons for the AIS marker phospho IκBα Ser32 (14D4) to identify the axon after surface immunostaining for HA-KCNQ3/KCNQ2 wild-type WT or mutant (A343D and R353G) was completed in Fig. 8B. Inverted images (lower) show the AIS marker phospho IκBα Ser32 (14D4) immunostaining in the insets of the GFP-transfected neurons (middle). Camera lucida drawings (upper) were constructed from the inverted gray-scale image of the GFP-transfected neurons (middle) in which the axons were traced in black. Arrows indicate the AIS. Scale bars are 20 µm. (TIF) [file pone.0103655.s004.tif]

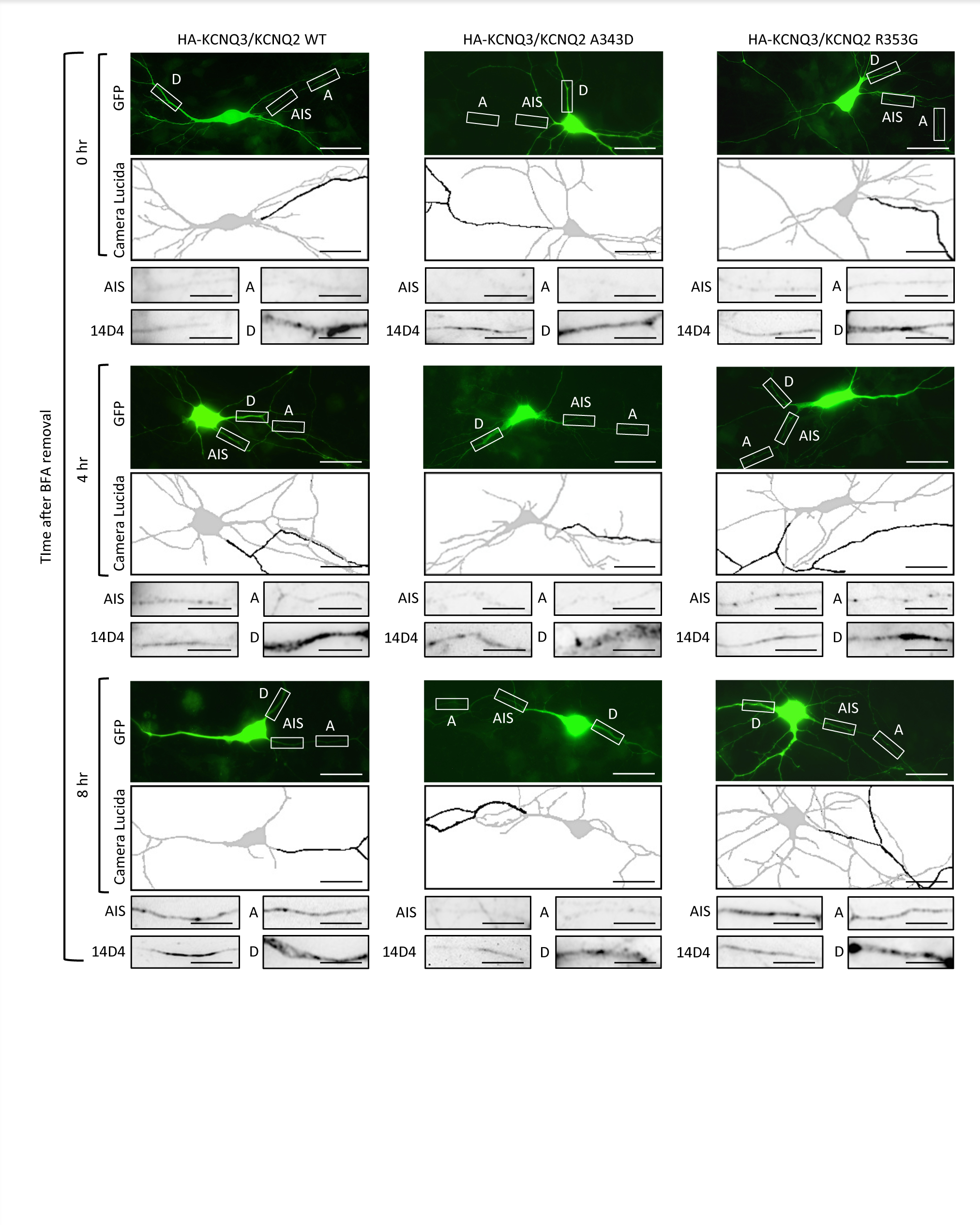

Supplement: Figure S5 — Pulse-chase assay of HA-KCNQ3/KCNQ2 channels from the ER. Representative images of wild-type (WT) or mutant (A343D and R353G) HA-KCNQ3/KCNQ2 channels at 0, 4, and 8 hr after BFA washout in hippocampal neurons cotransfected with GFP (green). The axon was identified by immunostaining with the AIS marker phospho IκBα Ser32 (14D4). Camera lucida drawings (middle) of the GFP-transfected neurons (upper) show the soma and dendrites (gray) and an axon (black). Scale bars in the upper and middle panels are 20 µm. The small lower panels are representative inverted images of HA-KCNQ3/KCNQ2 at the AIS (AIS), distal axons (A), and dendrites (D) in transfected neurons (insets). Scale bars of the small lower panels are 10 µm. (TIF) [file pone.0103655.s005.tif]
